# Efficiency Analysis in Brazil’s Sao Paulo State Local Unified Health System (SUS): From Gender-Ethnicity-Power Inequities to the Dissolution of Health Effectiveness

**DOI:** 10.3390/ijerph19052990

**Published:** 2022-03-04

**Authors:** Simone Schenkman, Aylene Bousquat, Maria Paula Ferreira

**Affiliations:** School of Public Health, Sao Paulo University, Av. Dr. Arnaldo 715, Cerqueira César, Sao Paulo 01246-904, Brazil; aylenebousquat@usp.br (A.B.); mpferrei@seade.gov.br (M.P.F.)

**Keywords:** health equity, efficiency, intersectionality, Unified Health System, capitalism

## Abstract

Health equity is cross sectioned by the reproduction of social relations of gender, ethnicity and power. The purpose of this article is to assess how intersectional health equity determines societal health levels, in a local efficiency analysis within Brazil’s Unified Health System (SUS), among Sao Paulo state municipalities. Fixed Panel Effects Model and Data Envelopment Analysis techniques were applied, according to resources, health production and intersectoral dimensions. The effect variables considered were expectation of life at birth and infant mortality rates, in 2000 and 2010, according to local health regions (HR) and regionalized healthcare networks (RRAS). Inequity was assessed both socioeconomically and culturally (income, education, ethnicity and gender). Both methods demonstrated that localities with higher inequities (income and education, gender and ethnicity oriented), associated or not to vulnerability (young and low-income families, in subnormal urban agglomerations), were the least efficient. Health production contributes too little to health levels, especially at the local level, which is highly correlated to the intersectoral dimension. Intersectional health equity, reinforced in its intertwining with ethnicity, gender and social position, is essential in order to achieve adequate societal health levels, beyond health access or sanitary and clinical efficacy.

## 1. Introduction

Health inequities must be analyzed from the angles of critical epidemiology and intersectionality, in a simultaneous, totalizing and dialectical manner. Therefore, a joint effort must be made to appreciate the richness contained in the transversality of gender, social class, occupation, ethnicity and spatial position in the social determination of health. Power relations permeate the entire framework that determines inequities in society, in an intersectoral perspective, overflowing their effects on the health levels [1,2,3].

In the case of gender and ethnic differences in the productive insertion, it is necessary to take into account the qualification and discrimination levels, which are reflected in the design of positions, salaries and institutional hierarchies, against a patriarchal and racist society, determining the gender-imposed role choices. It is known that the discrimination factors in the world of work are greater in relation to gender than to ethnicity, as the latter suffers a more profound type of discrimination, related to education and qualification, which has its roots in social and power relations of the historically constructed slave society [4,5]. The *ideal* society for the capitalist system to function is the patriarchal society, with the white man exercising control over others, including nature [6,7].

Incidentally, from the point of view of socioenvironmental determination, the capitalist accumulation system is characterized by exhibiting an oppression, exploitation and expropriation model, with consequences for health. The right to health becomes increasingly frail, with the commodification and privatization of essential services, previously provided by the public sector, increasingly fraying the social fabric on which it is anchored [8,9,10].

Intersectionality, on the other hand, is based on the understanding that we are shaped by the interaction of distinct social positions, which occur in an environment and power structure context, such as laws, policies, governments, social institutions, religion and media. Through these processes, independent forms of privilege and oppression are created, under colonial-imperialism, racism and patriarchy [11]. These relationships are expressed from the very conception of the right to health and its respective implemented health policies, to health access and actions, favoring or hampering its promotion, prevention and recovery [12]. Moreover, the time period required for inequity to manifest itself in population health levels would be of five years, with a peak at seven years and decreasing after twelve years [13].

In the perspective of understanding how inequities germinate in the Brazilian socioenvironmental space, and that of the state of Sao Paulo, we start out from its socioeconomic background, which is marked by a dependent model of capitalism. Some of its characteristics are the distancing of the internal productive structure from the workers’ collective needs, the workforce overexploitation and the premature wearing out of workers, with low wages and local taxes that are more advantageous to central economies [14].

Regarding the state of Sao Paulo, Brazil, which accounts for 31.93% of the Brazilian gross domestic product (GDP) [15], its development model, with decentralized industrialization since the 1970s, has brought many benefits, but also many contradictions. We point out that the cities of Campinas and Ribeirão Preto benefited from industrial and agro-industrial development in a more equitable model, bringing well-being to the countryside, with smaller agglomerations than those surrounding the capital city. Thus, the model developed in the metropolis was quite harmful to its surroundings, which, despite achieving high success rates in education, failed to distribute its income in an adequate manner. Those regions that grew in a complementary way to the capital city had its advantages, as long as they were not too close, where the model acquired serious contradictions and generated dependencies [16], including health care.

To analyze how the social determination of health is inscribed in the territory of the state of Sao Paulo, it is necessary to understand the configuration of the Brazilian Unified Health System (SUS, *Sistema Único de Saúde*) in networks and regions, aiming to guarantee the principles of universality, integrality and equity.

The Brazilian Health System is a dual system, with a public health system denominated SUS (its acronym in Portuguese), free and universal, which is 32 years old, and coexists with the private sector in a complex dynamic, with 75% of its population depending solely on the public sector. Despite its principles of universality, integrality and equity, it struggles with reduced health financing and a rather insufficient proportion of public expenditure, less than half of Brazil’s total health spending as a proportion of GDP of 8% [17]. The SUS, regardless of its chronic lack of funding, has greatly improved the population’s health conditions, ensuring increased access to health services altogether and specifically to primary care [18]. This is due to the fact that it offers an important proximity to the local population with a great amount of capillarity, especially in its primary health care services. It has also greatly increased ambulatory and specialized procedures, although being constantly threatened by private-public provision arrangements, which try to shift its healthcare model and do not relieve the public system. These arrangements depict the articulation between three complexes: the medical-industrial, the medical-financial and that of support services, contributing to further increase inequities in funding, access and utilization of health services [17].

The SUS regionalization process has been going through different phases since its implementation, which took place rather late and slowly, but with considerable advances. It is important to emphasize that the current phase derives from the implementation of Ordinance n. 4.279/2010 and Decree 7.508/2011, with successive changes in network and region conceptions, until they reached the health care networks in negotiated and contracted regions. This pact occurs between the government levels and brings greater flexibility and gains due to scale, with resources bounded to thematic networks, extrapolating health regions and reconfiguring them, aiming to reduce disparities in the provision of health services, but also bringing about the need for finer adjustments between levels [19].

In 2011, the criteria and strategies for the construction of regionalized health care networks (RRAS—*Redes Regionalizadas de Atenção à Saúde*; *n* = 17) were gathered in the state of Sao Paulo, based on the sufficiency of primary, medium and part of high complexity care; population between 1 and 3.5 million, economy of scale and maintenance of the territory of previously constructed health regions (HR; *n* = 63) and their assistance flows. They are characterized by their complex design, which combines homogeneous services in heterogeneous areas, considering thematic networks and the diversity of institutions and actors, in addition to conflicts between regions, fragmented in their equality or united in their differences, with major governance challenges [20].

Considering the different SUS network and region conceptions, we realize that the main socio-spatial and access inequities in health care were addressed; nevertheless, health levels inequities were completely disregarded. Therefore, the mismatches between efficiency, effectiveness and equity were thoroughly experienced during this process.

Moreover, the relation between these concepts is complex and specifically for Brazil, few studies on local health efficiency have been carried out. Nearly two-thirds of the efficiency studies performed globally [21] were about productive, technical or scale efficiency in health services. Furthermore, these kinds of efficiency analyses with the frontier methods seek a more organizational view, whereas effectiveness and allocative efficiency stand on a programmatic level. Very few studies were based on health systems (2%), and, to our knowledge, even fewer were developed on local health systems or on a systemic level, which includes health equity as a cross-sectional dimension.

As examples of well-founded studies at the local level, we point out that of Sousa et al. [22], who presented efficiency scores about general services in Brazilian municipalities; Marinho [23], who analyzed the efficiency of the provision of health services in municipalities in Rio de Janeiro; and Souza et al. [24], who sought to assess the productive efficiency of the public hospital sector in the cities of São Paulo. Stochastic and non-stochastic efficiency frontier methodologies were used, combined with regression models, as well as bootstrap and jackknife methods, in order to reduce the effects of outliers.

There are also studies focusing on primary health care (PHC), which attempted to differentiate the relative efficiency of health actions and health outcomes in Brazilian municipalities [25] and relate efficiency to the view of health professionals, in order to assess health promotion in selected municipalities in Minas Gerais [26].

In addition to the small number of Brazilian studies that specifically address health efficiency, in addition to simply testing the methodology or working particularly on productive, technical and scale efficiency, most of them address aspects more focused on health service outcomes and not health system outcomes at the local level.

The aim of this article, therefore, is to assess the role of equity in health in determining societal health levels, in a local effectiveness and efficiency analysis of Brazil’s SUS, among Sao Paulo state municipalities. Hence, we intend to strengthen the analysis of the association between social inequities and vulnerabilities and the effects experienced in health and in life, taking into account the particular organizational design of Sao Paulo state.

## 2. Method

In order to assess local health efficiency in a comprehensive equity-concerned framework, the following dimensions were considered: material and financial resources, products and services of health production (access, coverage and prevention) and intersectoral variables. This selection allows evaluating the efficiency of input resources at health levels, with technical and allocation efficiencies combined, as well as productivity in health (products and services in health) and effectiveness, associating health intermediate results to its final impacts [27]. The effect variables considered were life expectancy at birth (*LEB*) and infant mortality (*IM*), for all the statistical models.

The variable selected to measure inequity was chosen in a intersectional perspective, associated with inequity by gender and ethnicity, from a socioeconomic (overall and work income) and cultural (level of education) point of view. Along these lines, other intersectoral variables tested were inequity by ethnicity in the proportion of vulnerability and unemployment.

From the governance perspective, the municipality’s transparency index was evaluated, according to an instrument of the Federal Prosecution Ministry (MPF, *Ministério Público Federal*). Unlike most international panels, there is no solid set of indicators in this area, much less at the municipal level. From the environmental point of view, variables related to basic sanitation were tested, such as water supply, waste disposal and the existence of sanitary sewage.

The time period considered was ten years, with observations ranging from 2000 to 2010 for all municipalities, with data available for the studied variables. The divisions used were the RRAS (*n* = 17) and its respective Health Regions (*n* = 63) in the following locations: Great SP (ABC, Alto Tietê, Franco da Rocha, Mananciais, Rota dos Bandeirantes and Capital); Santos and Registro; Sorocaba; Bauru; Marilia; Presidente Prudente; Araçatuba and São José do Rio Preto; Ribeirão Preto and surroundings; Piracicaba; Campinas and São João da Boa Vista and Taubaté. These divisions allowed grouping the municipalities according to their health care structure and the possibility of gains in scale, historical and cultural heritage, with a greater diversity than merely geographical divisions, better discriminating the differences between groups.

The techniques applied were the Fixed Effects (FE) panel data model and the Data Envelopment Analysis (DEA) in a dynamic and network slack-based model [28], in addition to a correlation analysis between methods. The chances of improvement in the final results and their impact on health are presented, such as the potential years of life gained and the reduction in *IM* rates, referenced by the municipalities that were benchmarks for efficiency. It is important to note that, while the FE model has a dual utility, of presenting an effectiveness analysis, in a temporal fashion, coupled with efficiency (residuals), the DEA, particularly the dynamic and network model used, allows for assessing efficiency, considering all stages of the production of health, in a dynamic time-driven manner.

The FE model admits the following premises: each locality has its own characteristics that could influence the dependent variables; some aspects of the unit can bias or weaken the interpretive power of the variables and control for this effect is mandatory. The FE model controls the time-invariant attributes of the independent variables, so that it permits a net effect interpretation. The locality specific effect was obtained by the sum of the municipality fixed effects and its residuals, which apply to inefficiencies at its upper limit, softening the effect of unknown variables and errors in measurement [29]. Another relevant assumption is that these unique time-invariant attributes are specific to the municipalities and do not correlate among them. Stata SE 10.1 software was selected to perform the analysis of the fixed effects panel models.

The following equation allowed for the complete models:

$$Y_{it}=\beta_{0}+\beta_{1}X_{1,\;it},+\cdots +\beta_{k}X_{k,it}+\gamma_{2}\varepsilon_{2}+\cdots +\gamma_{n}\varepsilon_{n}+\delta_{2}T_{2}+\delta_{t}T_{t}+\mu_{it}$$

where:

– Y*_it_* is the dependent variable (DV) where *i* = unit and *t* = time;
– X*_k,it_* represents the independent variables (IV);
– *β_k_* is the IV coefficient;
– *μ_it_* is the error term;
– *ε_n_* is the *n* unit;
– *γ*_2_ is the unit coefficient;
– *T_t_* is the time;
– *δ_t_* is the time-related coefficient.

For each dependent variable, the bivariate association with the explanatory variables, in addition to the multivariate model—by dimension and for the complete set—was obtained by means of the FE method. Additional tests were performed: the Hausman test, which permits to contrast the FE with the random effects model, and the heteroscedasticity assessment of variance constancy (when confirmed, we calculated the robust standard error or applied the bootstrap technique). The next step was to obtain the specific effects for each municipality, as the sum of its fixed effects and residuals, as a post hoc analysis. The complete model includes the efficiency gains values, benchmarked by the most efficient municipalities. 

The main advantage of the network DEA model is its multiple stages analysis, which surmounts the usual restrictions of the static models and leads to a greater correspondence to actual systems [30]. Consequently, in order to be considered efficient, the decision-making unit (DMU), must be efficient in all of its production phases; moreover, this technique enables revealing inefficiencies in a non-radial mode. This method assumes intermediate products to be produced and consumed inside the DMU, whereas inputs and outputs are external to its structure. The network model offers efficiency indices calculated at each point, besides the overall efficiency indices. The MaxDEA 8 Ultra software was chosen for the network slack approach.

The output-oriented model, with *k* stages, was considered, with the following equation:

$$\frac{1}{\tau_{0}^{*}}=max\;\Sigma_{k=1}^{K}W^{k}⟦1+\frac{1}{r^{k}+\sum_{h\;\in \;F_{k}}t_{k,\;h)}}\left(\frac{\sum_{kr=1}^{r}s_{r0}^{k+}}{y_{r0}^{k}}+\frac{\Sigma_{h\;\in \;F_{k}\;s_{h0}^{\left(k,h\right)+}}}{z_{h0}^{\left(k,h\right)}}\right)⟧$$

Subject to:

*z_o_*^(*k*,*h*)^ = Z^(*k*,*h*)^ *λ^k^* − *s_o_*^(*k*,*h*)+^

*Z*^(*k*,*h*)^ *λ^h^* = *Z*^(*k*,*h*)^ *λ^k^*

*s_o_*^(*k*,*h*)+^ ≥ 0

where *W^k^* is the relative weight of each section, *F_k_* is the set of phases with connection points (*k*, *h*), ∑*^K^_k_*_=1_ *W^k^* = 1, *W^k^* ≥ 0; *s^k^*^+^ are the product slack vectors, *r^k^* is the number of outputs at point *k*, *t*_(*k*,*h*)_ is the number of intermediate results in the connection between phases *k* and *h*, *s_o_*^(*k*,*h*)+^ slack vectors at the connections and *z* stands for the intermediate products.

For *LEB* (*n* = 645 DMU), the resource variables considered were average income and proportion of vulnerability (IPVS6 and population with less than ¼ of the minimum wage) and those related to inequity were measured by means of education and income (general and work) differentials, by gender and ethnicity; as intermediate products, we selected breast cancer screening, the proportion of newborns whose mothers had at least seven prenatal visits and the proportion of Caesarean sections. We also tested intersectoral variables, such as aging, unemployment and illiteracy rates, as well as the proportion of adolescent mothers and water supply. In the case of *IM* (*n* = 645 DMU), the same variables were tested, in addition to the proportion of children aged zero to five years out of school and people without electricity access. 

After analyzing the health impacts derived from the efficiency models through the potential years of life gained and the reduction in infant mortality rates, we proceeded in relating them to the levels of different aggregate variables, such as health and education levels, opportunities, living conditions and social vulnerability and equity. Further detailed analyses were performed with the visualization of São Paulo state maps, displaying the different levels of the effect variables, among regions and municipalities, as well as the health impacts obtained through both methods and the equity variables.

Spearman’s non-parametric correlations tests were performed to compare the techniques, regarding impact results for both dependent variables. 

## 3. Results

### 3.1. The Statistical Models: The Effects on Health Levels

The results obtained through the bivariate analysis of the FE panel model demonstrate that the financial resources had more significant results than the physical ones. The health production dimension, on the other hand, had little participation in determining the results for the set of municipalities.

In order to decide which variables were to be tested for the distinct dimensions, as well as the complete set model, we selected the relevant variables from the bivariate analysis (Table 1). The aggregate results in Table 2 depict that gender-ethnicity inequities in education, as well as income distribution, unemployment, illiteracy and aging rates, were significant for both effect variables in the intersectoral dimension, with the latter remaining in the final model of the *LEB* variable, together with the social vulnerability and environmental variables, such as the proportion of adolescent mothers. On the other hand, the *IM* variable included ethnicity and work-related income inequalities, in association with social vulnerability and intersectoral dimension variables, such as the proportion of children out of school. Both models reached high values of R^2^, close to 80%. 

A relevant finding related to the financial resources dimension is that the GDP per capita and the density of nurses proved to be determinants of *IM*, whereas only the vulnerability variables were important for *LEB*. Regarding health production, screening for breast and cervical cancer, in addition to the proportion of Caesarean sections and of newborns whose mothers had at least seven prenatal consultations, were decisive for both effect variables, but with a very low R^2^, of around 5%. In the environmental dimension, water supply and electricity access appear, respectively, as relevant variables for *LEB* and *IM*. Regarding governance, it is worth noting that the variable related to transparency did not remain in the final models. Among the dimensions, the intersectoral one showed the highest R^2^ values, close to 80%, followed by physical and financial resources, close to 70%. We should emphasize that some variables were significant only at the bivariate analysis, not persisting in the final models, such as *per capita* health expenditures derived from local tax revenues, the proportion of GDP related to services, the Gini index and the distribution of income by quintiles, dependency ratio, proportion of the population dependent on the elderly, proportion of the population with at least one disability, variables related to the Municipal Human Development Index (IDHM, *Índice de Desenvolvimento Humano Municipal*) and its components, in addition to those related to social responsibility (IPRS, *Índice Paulista de Responsabilidade Social*). For a complete view of all variables tested in the bivariate analysis, please consult the Supplementary Tables S1–S3.

### 3.2. A First Glance at the Health Impacts Derived from the Models

The final models equipped us with the health impacts and offered us a first glance at the potential years of life gained and the reduction in the infant mortality rates, across the regions.

**Figure 1:**
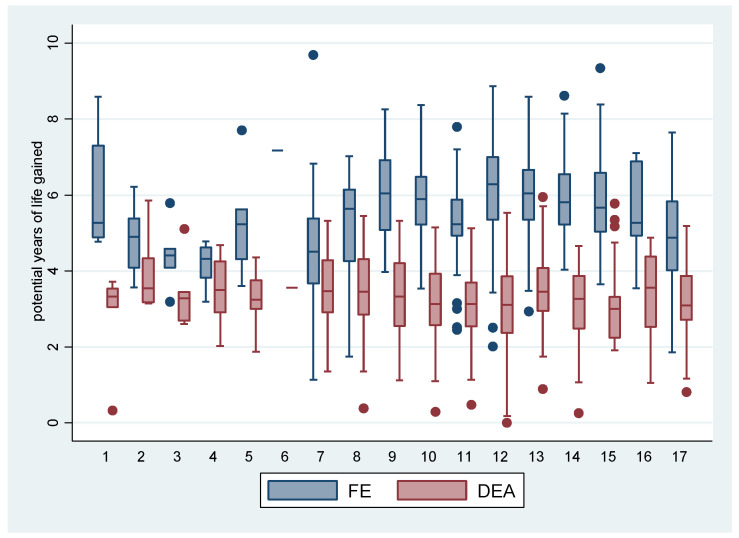
Dispersion of the impact on Life Expectancy at Birth, denoted by the potential years of life gained: contrasts between methods (FE and DEA), according to the Regionalized Healthcare Networks (RRAS). Note: The municipalities with the highest potential gains are Santos (RRAS 7), Nova Odessa (RRAS 15) and Bady Bassit (RRAS 12) and the benchmark municipalities are Eldorado (RRAS 7); Itapirapuã Paulista (RRAS 8) and Jambeiro (RRAS 17), as they express higher average levels of effectiveness and efficiency. **RRAS:** Greater SP (1. ABC, 2. Alto Tietê, 3. Franco da Rocha, 4. Mananciais, 5. Rota dos Bandeirantes and 6. Capital); 7. Registro and Santos; 8. Sorocaba; 9. Bauru; 10. Marília; 11. Presidente Prudente; 12. Araçatuba and São José do Rio Preto; 13. Ribeirão Preto and surroundings; 14. Piracicaba; 15. Campinas and São João da Boa Vista; 16. Campinas (Bragança and Jundiaí) and 17. Taubaté.

Figure 1, below, shows the potential years of life gained in *LEB* according to the regionalized health care networks (RRAS). It can be observed that the distributions obtained according to the FE model were more rigorous and showed higher values than in the DEA model, including more localities displaying extreme values (outliers). It is noteworthy that the *Mananciais* region (RRAS 4) exhibits proximate distributions for both methods.

**Figure 2:**
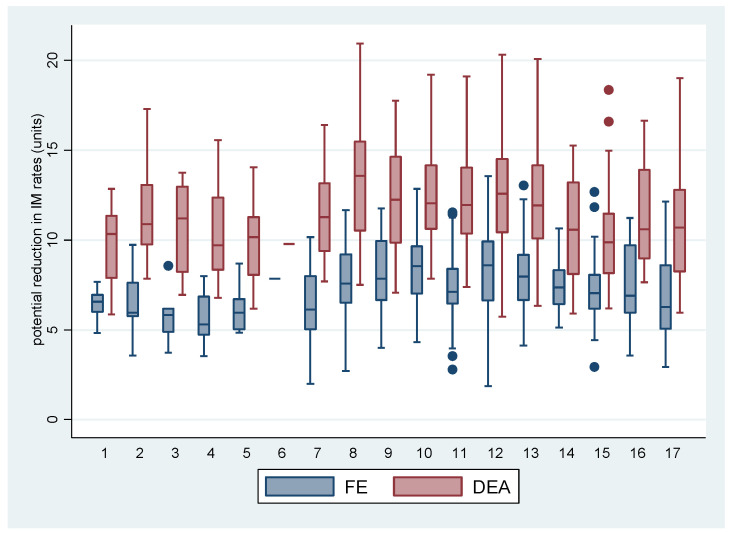
Distribution of potential reduction in the Infant Mortality rates: comparison between methods (FE and DEA), according to Regionalized Healthcare Networks (RRAS). Note: Additional municipalities with greater potential to reduce their *IM* levels were found in RRAS 8 (Barra do Chapéu), RRAS 9 (Uru and Coronel Macedo), RRAS 17 (Canas) RRAS 10 (Timburi), RRAS 12 (Mira Estrela and Gabriel Monteiro) and RRAS 13 (Altair and Trabiju). Jambeiro and Eldorado, in the opposite direction, remained as reference municipalities, in addition to Novais and Cedral (RRAS 12), Mirante do Paranapanema (RRAS 11), Águas de São Pedro (RRAS 14), Cosmópolis (RRAS 15) and Ilhabela (RRAS 17). **RRAS:** Greater SP (1. ABC, 2. Alto Tietê, 3. Franco da Rocha, 4. Mananciais, 5. Rota dos Bandeirantes and 6. Capital); 7. Registro and Santos; 8. Sorocaba; 9. Bauru; 10. Marília; 11. Presidente Prudente; 12. Araçatuba and São José do Rio Preto; 13. Ribeirão Preto and surroundings; 14. Piracicaba; 15. Campinas and São João da Boa Vista; 16. Campinas (Bragança and Jundiaí) and 17. Taubaté.

Figure 2 then shows the potential reductions in the *IM* rates for the RRAS. Unlike *LEB*, the distributions showed higher values in the DEA model than in the FE model. 

The best results, according to the FE model for both effects (*LEB* and *IM*) were observed in Greater SP (Mananciais RRAS 4), in Registro and Santos (RRAS 7) and Taubaté (RRAS 17). The worst ones are in the capital, regarding life expectancy (RRAS 6) and in Marilia, for infant mortality (RRAS 10). This is due to an imbalance in the distribution of wealth and education in these places, with the worst inequity results, even with high wealth (SP), or proportionally with less wealth, but also with an unequal distribution, especially in relation to education, reflected both in the high rates of illiteracy and in gender-ethnicity inequities (Marilia). With the DEA model, the best results for life expectancy were found in Campinas (RRAS 15), while the capital had the best results for the infant mortality rate, while the worst results in life expectancy were found for the Alto Tietê region—RRAS 2 (high inequity and low reach)—and the worst infant mortality rates were found in Sorocaba —RRAS 8 (worst rate of *IM* and average inequity).

When the stages of health production in the DEA methodology were analyzed, there was a substantial reduction in efficiency (12–30 p.p. percentage points), demonstrating that health care did not contribute to the final health results as expected. When considering the health production variables, a lot of efficiency is lost and, in this case, the best results are from Presidente Prudente in relation to *LEB* (best result in breast cancer screening) and from the capital for the *IM* rate (reduced *IM* rate, as well as illiteracy and children aged 0 to 5 years out of school), whereas the worst ones are in the capital, this time for *LEB* (high levels of inequity and vulnerability do not allow the attainment of efficiency, even with all the wealth achieved) and Araçatuba, for the *IM* rate (high rate of illiteracy and proportion of Caesarean sections).

The efficiency averages were generally higher for *LEB*, with smaller disparities than the *IM* rates. Considering the discrepancies in the efficiency indices between the effect variables, they ranged up to 40 p.p. between the two variables in some locations. Regarding the regions (RRAS), Araçatuba and Ribeirão Preto and adjacent areas were the ones with the smallest differences, followed by Sorocaba and Bauru. At the opposite direction, Greater Sao Paulo (especially Mananciais) had the biggest differences.

### 3.3. Putting It All Together: Further Views and Synthesis

After analyzing the differences between regions and its rankings on both health impacts and selected aggregate variables (Table 3), we verified five distinct typologies for the regions. First of all, the least efficient locations are those that, although presenting better levels of health, living conditions and education, exhibit high social vulnerability, medium opportunities and large inequities in income and education by gender and ethnicity, such as the capital city of SP (i) and part of its surroundings (spreading into Santos and Sorocaba and sparing the Mananciais and Franco da Rocha region). These regions, in comparison to the capital, have worse education levels and living conditions but higher equity levels (ii—Santos and Sorocaba), or worse social vulnerabilities and opportunities but higher equity and education levels (iii—Franco da Rocha and Mananciais). The central region of the state and the western fringe (iv) also showed great potential for improvement in efficiency; exhibiting worse levels of living and health conditions, but with less inequity of income and vulnerability and better opportunities, but with worse levels of education, with inequities regarding gender and ethnicity (ranging from Sorocaba to Bauru/Marília; from Piracicaba; to some of the surrounding health regions of Ribeirão Preto, Araçatuba and São José do Rio Preto). On the contrary, the last typology (v) includes the south vector, especially to the southeast (medium health levels), and the northwest vector, especially to the west (lower health levels), which show a better balance between these variables, as well as the region of Campinas and Ribeirão Preto (higher health levels).

For a complete view of the impacts in the health levels, based on the efficiency models performed in all Health Regions and Regionalized Health Networks, in detail, please consult the Supplementary Figure S1.

We gathered that in all RRAS, the municipalities that show the lowest efficiency rates and, therefore, the greatest potential for improvement, are those with the highest level of vulnerability or inequities, in addition to the existence of conflicts, toxic economic development models, workers’ overexploitation and environmental disasters, showing an equivalence between the statistical methods employed.

**Figure 3:**
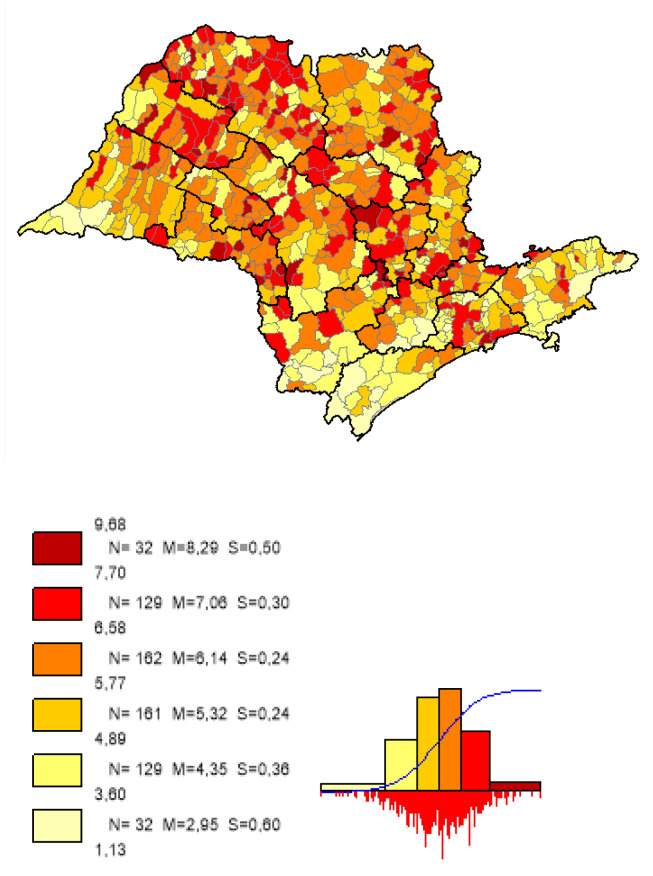
Distribution map of potential years of life gained in municipalities in the state of SP, fixed effects. Note: Choropleth maps, consisting of six divisions, using the following limits—minimum value; 5th percentile; 25th, 50th and 75th percentiles; 95th percentile and maximum value. For each interval, the number (N) of municipalities, their mean (M) and standard deviation (S) are described. The histograms contain bars proportional to the number of localities in each division (*Philcarto* images).

**Figure 4:**
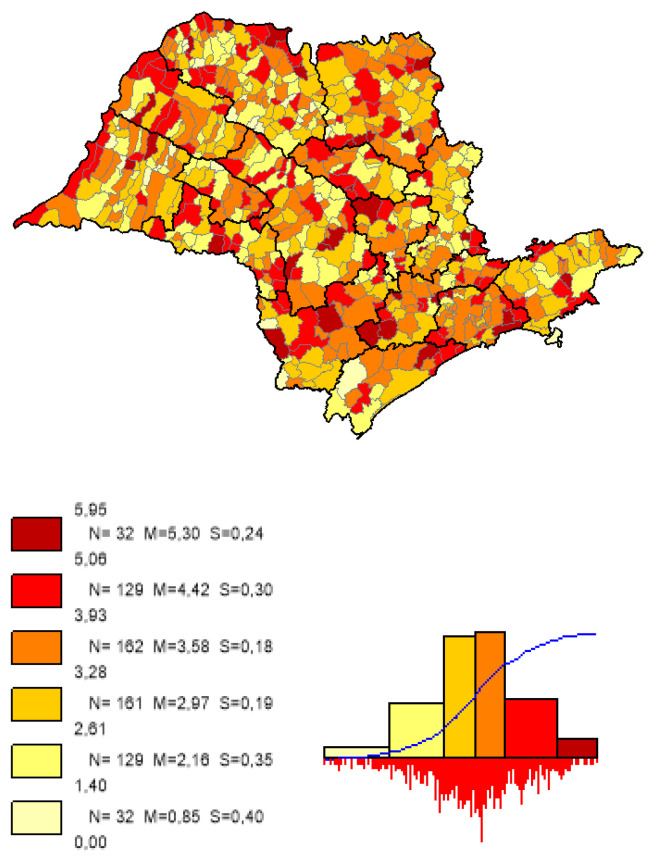
Distribution map of potential years of life gained in the municipalities of the State of Sao Paulo, DEA method. Note: Choropleth maps, consisting of six divisions, using the following limits—minimum value; 5th percentile; 25th, 50th and 75th percentiles; 95th percentile and maximum value. For each interval, the number (N) of municipalities, their mean (M) and standard deviation (S) are described. The histograms contain bars proportional to the number of localities in each division (*Philcarto* images).

**Figure 5:**
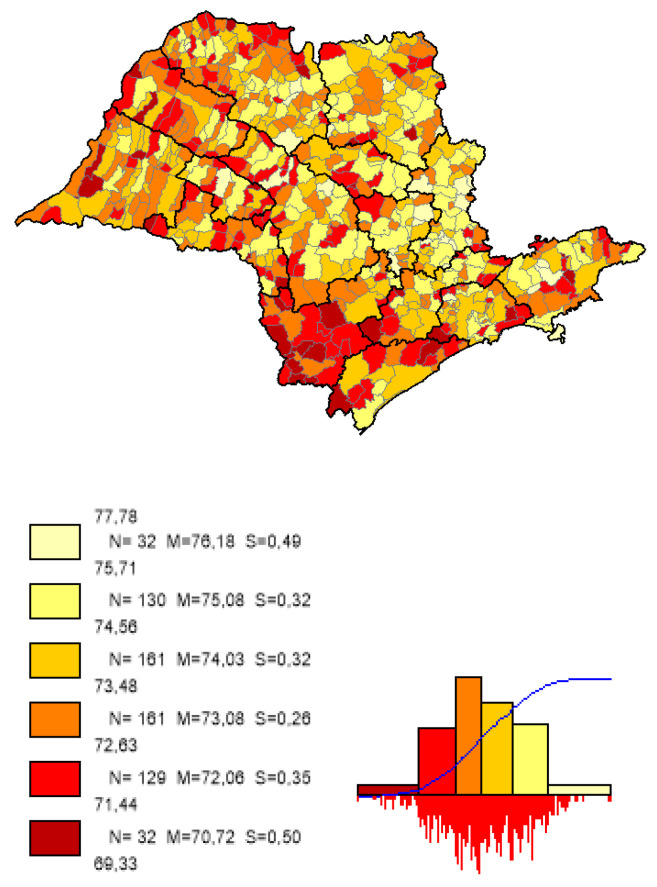
Distribution map of Life Expectancy, average results in the municipalities of the state of SP. Note: Choropleth maps, consisting of six divisions, using the following limits—minimum value; 5th percentile; 25th, 50th and 75th percentiles; 95th percentile and maximum value. For each interval, the number (N) of municipalities, their mean (M) and standard deviation (S) are described. The histograms contain bars proportional to the number of localities in each division (*Philcarto* images).

**Figure 6:**
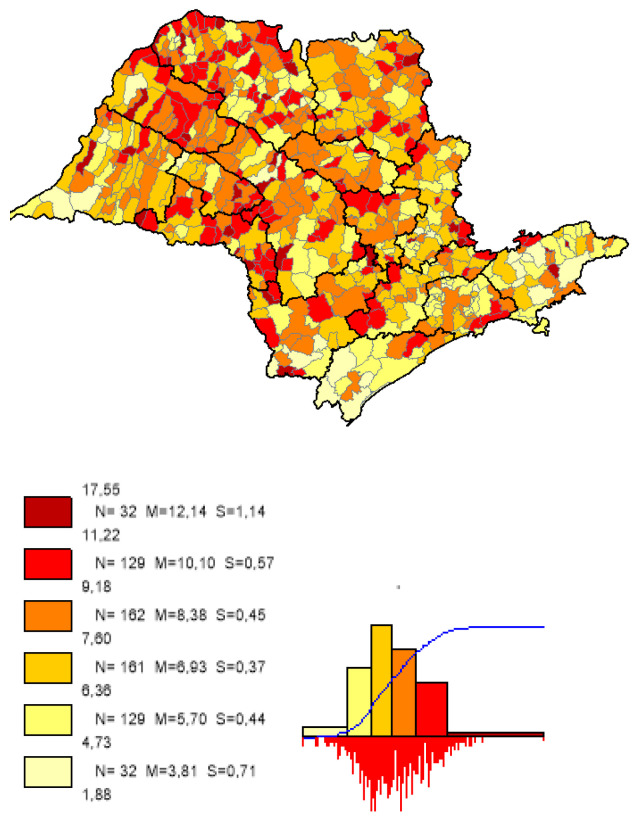
Distribution map of the potential reduction in *IM* rates in the municipalities of the state of SP, fixed effects. Note: Choropleth maps, consisting of six divisions, using the following limits—minimum value; 5th percentile; 25th, 50th and 75th percentiles; 95th percentile and maximum value. For each interval, the number (N) of municipalities, their mean (M) and standard deviation (S) are described. The histograms contain bars proportional to the number of localities in each division (*Philcarto* images).

**Figure 7:**
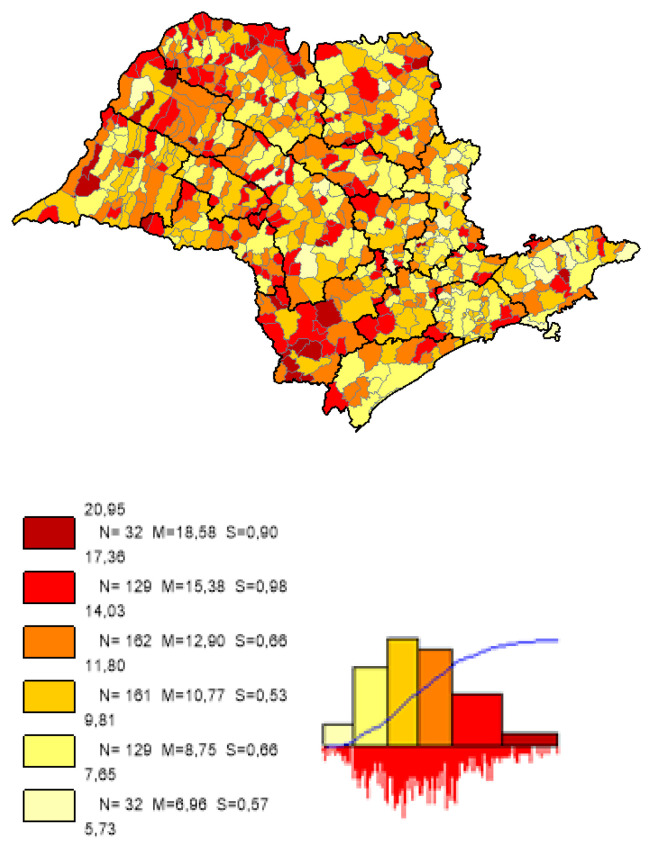
Distribution map of the potential reduction in the *IM* rates in the municipalities of the state of SP, DEA method. Note: Choropleth maps, consisting of six divisions, using the following limits—minimum value; 5th percentile; 25th, 50th and 75th percentiles; 95th percentile and maximum value. For each interval, the number (N) of municipalities, their mean (M) and standard deviation (S) are described. The histograms contain bars proportional to the number of localities in each division (*Philcarto* images).

**Figure 8:**
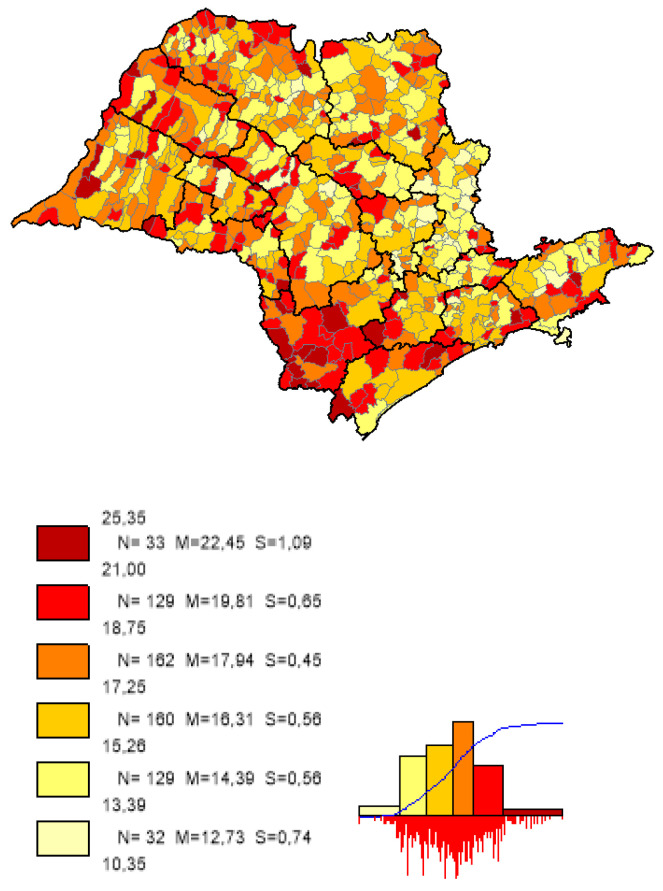
Distribution map of *IM* rates average results in the municipalities of the state of SP. Note: Choropleth maps, consisting of six divisions, using the following limits—minimum value; 5th percentile; 25th, 50th and 75th percentiles; 95th percentile and maximum value. For each interval, the number (N) of municipalities, their mean (M) and standard deviation (S) are described. The histograms contain bars proportional to the number of localities in each division (*Philcarto* images).

We can observe based on the constructed maps that the distributions of the potential years of life gained (Figure 3 and Figure 4) are inverse to the average *LEB* results (Figure 5); that is, in the opposite direction to the expected one. The opposite happens to the distribution of the potential reduction in the *IM* rates (Figure 6 and Figure 7) and its average results (Figure 8).

**Figure 9:**
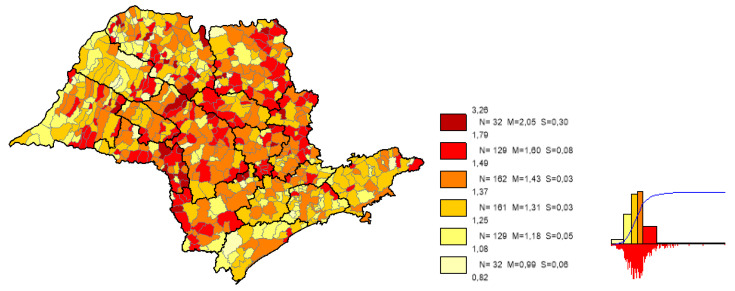
Distribution of gender-ethnicity educational inequity, in the municipalities of the state of SP. Note: Choropleth maps, consisting of six divisions, using the following limits—minimum value; 5th percentile; 25th, 50th and 75th percentiles; 95th percentile and maximum value. For each interval, the number (N) of municipalities, their mean (M) and standard deviation (S) are described. The histograms contain bars proportional to the number of localities in each division (*Philcarto* images).

**Figure 10:**
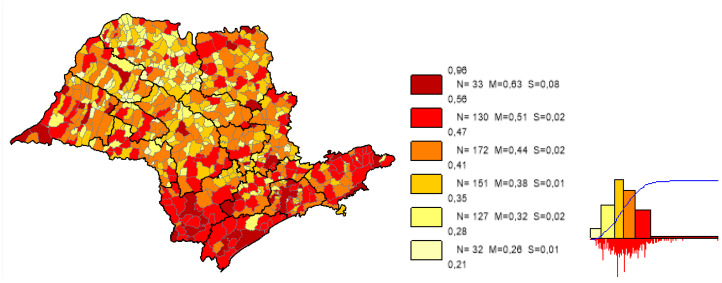
Distribution of inequity regarding overall income distribution (Theil index), in the municipalities of the state of SP. Note: Choropleth maps, consisting of six divisions, using the following limits—minimum value; 5th percentile; 25th, 50th and 75th percentiles; 95th percentile and maximum value. For each interval, the number (N) of municipalities, their mean (M) and standard deviation (S) are described. The histograms contain bars proportional to the number of localities in each division (*Philcarto* images).

**Figure 11:**
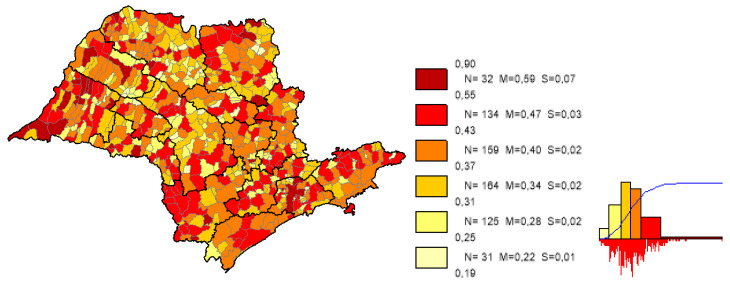
Distribution of inequity regarding work income distribution (Theil work) in the municipalities of the state of SP. Note: Choropleth maps, consisting of six divisions, using the following limits—minimum value; 5th percentile; 25th, 50th and 75th percentiles; 95th percentile and maximum value. For each interval, the number (N) of municipalities, their mean (M) and standard deviation (S) are described. The histograms contain bars proportional to the number of localities in each division (*Philcarto* images).

Most strikingly is the fact that the lower the inequities, the greater the efficiency (Figure 9, Figure 10 and Figure 11), as shown in the inequity maps of the level of education by gender and ethnicity and the overall and work income Theil index. The intersectional educational inequities exhibit a very close resemblance to the Fixed Effects impacts for both effects (Figure 3 and Figure 6); as for the Theil index distributions, we may note a closer similarity to the DEA results (Figure 4 and Figure 7).

We also tested the correlations between the results of the applied methods (FE and DEA): for the variable *LEB*, the results were only satisfactory, **0.532**. For the *IM* rate, this correlation was significantly higher, reaching **0.803**.

## 4. Discussion

### 4.1. The Models and Techniques: What Do They Tell Us?

Health equity, from a critical perspective, is essential in order to attain good final results for society. Whereas the balance between wealth and its equitable distribution undoubtedly influences health efficiency, its most relevant aspect lies within the tripartite equity, weaving the categories of social class, gender and ethnicity [1], especially in comparisons under the same general framework, such as the local health system analysis we performed. 

The municipalities with the best health results were not always the most efficient ones. This occurred exactly because of the gap regarding the variables related to equity, especially the difference in education between gender and ethnicity. This is a more structural variable than simple income differences [4], such as Theil’s indexes tested, overall and work-related income. It is noteworthy that intersectional equity did not achieve the same importance in our previous analysis of efficiency at the global level [31], which allows us to assume that the local level is the ideal *locus* for these differences to gain visibility.

In our models, health care did not explain efficiency enough, which showed a high association with the intersectoral dimension: this is because municipalities are being compared within the same health system. Although exhibiting loco-regional differences, they were not differentiated in the model, with the healthcare variables tested. This may also be due to not assessing healthcare utilization according to the populations’ needs, which would maybe unveil these differences on a local scale [32,33].

On another note, we did not find a relationship between financial resources and health levels in the overall models; on the contrary, they were strongly related to the dimension of health production (*per capita* local health revenues and expenditures) or were only present in the dimension of resources for the *IM* variable (*per capita* GDP). However, inequity and vulnerability were strongly related to health levels. In this sense, Biggs et al. [34] indicated that the positive association between material living conditions (*per capita* GDP) and health levels revealed itself only when reduction or stabilization of poverty and inequity were also present, interposed by *LEB* and *IM*, demonstrating they were effect modifier variables.

The differences observed in the results were due to the fact that the FE method showed an imbalance in the distribution of wealth and education in the assessed locations, whereas in the DEA, the differences were due to the wealth and results already achieved in large cities, which are more valued by the method, even with high levels of inequity. Therefore, they should both be used, as they depict different complementary aspects, especially at the local level.

### 4.2. Examples of Economic Development Models in Sao Paulo

The state of Sao Paulo produced a unique invention in its decentralization and regionalization process, in the sense of integrating the concepts of network and region in a RRAS, promoting the integrality of health care units, with universality and equity at its base. The problem is that the territory brings inequities and social dynamics, which are not always incorporated into the planning or its selected delimitations. Furthermore, it involves multiple actors and institutions: the state, society and the market, with particular interests. Therefore, there are many extreme values in the analyzed distributions, as the RRAS were not conceived for their equitable homogeneity, but for geo-economic and political-administrative issues, in addition to sociocultural identities and the sharing of infrastructure, communication and logistics networks [19,35].

The diverse historical and economic development, as well as the predominant economic activity in the most efficient regions show great variations and we should concentrate and examine a couple of examples, in order to illustrate the results. It is above all important to highlight the contradictions found, such as: (i) the promotion of arts, culture and ecological tourism in the region of Mananciais, coexisting with a high proportion of districts with very high vulnerability and a large proportion of the population that receives less than ¼ of the minimum wage, housing shortage, deforestation and irregular settlements and rural regions; (ii) the eco-forest system activity in Registro and the diversified economic development centers in Taubaté, using medium-high technology; and (iii) the large offer of formal jobs in the Oil and Gas sector in Santos, but also surrounded by environmental conflicts, related to the expansion of the port area and the pre-salt exploration.

The region of Franco da Rocha (RRAS 3) should also be underlined, as it has the highest equity and scored well in efficiency, close to Mananciais, mainly due to reaching both the lowest ethnicity income ratio and the Theil index related to work income. It is a region with tourism and secondary residences (Mairiporã), extractivism (Cajamar and Franco da Rocha) and cellulose (Caieiras), but with high social vulnerability and many environmental disasters, such as floods and landslides and precarious infrastructure and access (Francisco Morato, pendulum city) and with a historical and symbolic molding related to the Juquery Psychiatric Hospital (Franco da Rocha).

It is also important to emphasize that the Araçatuba region had the best result regarding the Gini and Theil indexes, as well as in vulnerability (low proportions of districts with very high vulnerability), followed by the Presidente Prudente region, which shows, however, lower efficiency results, mainly due to the high illiteracy rates. The Campinas and Ribeirão Preto regions showed intermediate efficiency results, considering the high wealth and medium distribution, with urban agglomerations resulting from their long and sustained growth and concentration of transport, logistics and economy of scale. They have also suffered some depletion of the capacity to radiate well-being, as originally planned by regional development policies. These contradictions between appearance and essence of the capitalist mode of production lead to the deterioration of the environment and manifest themselves in cognitive, social and economic ways [36].

### 4.3. The Typologies: On How Social Inequity and Vulnerability Interplay in the Context of Capitalism

The different typologies observed in the state of Sao Paulo depict how the structural causes are important in determining the health levels. We verified a strong imbalance between health and education levels and living conditions on one side, and opportunities, vulnerabilities and social equity on the other. It is clear from our results that social sectors should be organized in such a way as to improve this balance, rather than having its design based solely on administrative proximities.

We have found greater potentials for life expectancy improvements and infant mortality reductions exactly in those more developed localities that reached high average health levels, but based upon income and work inequities and social vulnerabilities or on those less developed countryside localities with lower health and education levels and living conditions, but with less social vulnerabilities and more opportunities. Our great concern is that these localities, although with less overall inequities, present themselves with more structural inequities, such as gender and ethnicity on education and income levels.

It is crucial to understand how the concept of social vulnerability is inscribed in the dynamics of capital, with the dominance of fictitious capital and green economy, along with the large growth of the service sector, constraining the spatial circuits of socioeconomic production. According to critical geography [37], the superior circuit (of the owners and holders of the means of production) subsists on the inferior one (working class), which submits for fear of falling back into the sphere of complete exclusion. As we have described, social vulnerability is all the more present in the localities with the greatest health levels and living conditions, though badly distributed, or the medium levels of these dimensions, but with a greater proximity to the central regions, especially the capital. In that sense, we can observe two different situations: being close to the capital and having at least the benefits of higher education levels, although not of income, and that of being depleted on education and income levels, with important overall and work income inequities, which is the worst scenario.

At the same time, the socio-environmental relationship is weakened by the intensification of use and depletion of the soil *and workers*, exposed to pesticides and forced to abandon traditional and ecological forms of production in the countryside, to favor a brutal model of indifferent extractivism and the commodification of what was obtained naturally from the soil, in the past [38,39]. Likewise, the health sector commodification intensifies social vulnerability, as what was offered in the past to workers to improve quality of life, has been decreasing greatly [10], encompassing a slower growth on health levels and a poorer distribution of its benefits to society [40].

The pendular movement of the Brazilian internal bourgeoisie also helps us understand this dynamic of imperialist capital, occupying an intermediate position between the former national bourgeoisie and the buying bourgeoisie class, associated with imperialism. Their social position oscillates between dependence and contradiction with foreign capital, accepting the subordinate place in the international division of labor and always keeping their interests, above all, on the expanded reproduction of capital, without any socialization of risks, despite accepting to be dependent on the State and public funds [41]. The relation between social position and inequities found in our study were mainly due to ethnicity-oriented income inequities, overall and from work.

Currently, the productive capital is an increasingly smaller part of the system, dominated by financial capital, mainly the fictitious one, which, by the hand of the market and the State, makes promises to investors through financial collateral arrangements and public debt bonds, expropriating the workers’ future. According to Fontes [42], “the flight forward through State indebtedness, on a delusional scale, means the commitment of the States themselves in a direct manner, with the exacerbation of the future extraction of value”. Thus, if currently the premature exhaustion of the workforce does not imply a decrease in the average life expectancy, the quality of life and health has dropped considerably, as well as the full consumption capacity, generating situations of social vulnerability [14]. Our results embrace this concept, as we have noted that the highest average results in life expectancy occur exactly in the most unequal localities, along with high levels of social vulnerability.

Social vulnerability must be understood within its historical and social dimensions, as a deprivation of the power to act, to demand the guarantee of social rights and to take care of one’s health, within a logic of relations of oppression, domination, stigmatization, manipulation and exclusion, with socioeconomic and civil aspects. Moreover, it is accompanied by an ideology of blaming the socially frail, without taking into account their capacity for resistance and change, cross-sectioned by gender, ethnicity and social class relations [12,43]. We have found that gender and ethnicity related inequities in educational levels and income inequities (general, ethnicity and work related) affect local health efficiency greatly.

Within the logic of serving the financial capital and justifying fiscal adjustment measures, regardless of social vulnerability, an efficiency study was carried out by the World Bank [44], using the DEA methodology to evaluate the efficiency of Brazilian municipalities according to the levels of attention and the macroregions. The key finding, that PHC is efficient in terms of productivity but not performance, demonstrates the underutilization of its potential to improve equity. Moreover, it contends that the secondary and tertiary levels are less efficient, due to the small hospitals, which do not have an economy of scale, but are located in more remote regions, promoting equity in access. These considerations show the contradiction of the study that proposed a fair adjustment, with equity and efficiency regarding public spending. The analyses were not controlled by intersectoral and cross-sectional equity variables, and it was finally proposed to increase PHC productivity, with more procedures, reducing the costs with small hospitals, totally contrary to the sense of universal access and integrality, which go much beyond the procedures and the economy of scale. The lack of an equity adjusted model and of an effectiveness-oriented analysis, failed to produce a fair adjustment altogether.

In contrast, in our study, both methods showed that the municipalities with the highest degrees of inequities (in terms of income and education, by ethnicity and gender), associated or not with social vulnerability (young, low-income families, in subnormal urban agglomerations) exhibited the lowest efficiency indices, not attaining its full potential for health effects. We would like to point out that extreme poverty and selective social policies, particularly for the health and education sectors, have also been established as essential causal mechanisms in the association between social inequity and health levels [45], as well as livelihoods and structural, material and symbolic conditions, especially the educational level, acting independently, going far beyond the *per capita* income, at the local and global level [46].

Therefore, we must look for new ways to measure the final impact results on people’s lives and health. Far from the usual measures of quality of life or happiness, we need new measurement tools that can apprehend the contradictions, contrasts, wearing out and, above all, the sinuous cross-sectional movements within the categories in which we are all stratified [11].

## 5. Study Limitations

It was not possible to assess the governance variables, except for the MPF transparency index. For a future study, a proper way to do so would be to study the effectiveness of the action of municipal health councils in the monitoring and inspection of health actions, which would be a more objective way of evaluating governance and the prevention of corruption in the health area [47].

There are very few Brazilian studies on efficiency at the municipal level, and the few that have been carried out were related to efficiency in public management in general [22] or related to the technical efficiency of hospital health services [23,24] or primary health care [25,26]. What we proposed was to define the efficiency of local health systems, which was in part hindered by the fact that all municipalities belong to the same health system. On the other hand, due to local heterogeneities, regarding the intersectional aspects, it was possible to apprehend differences in social inequities and vulnerabilities.

As much as the authors have tried to encompass the different dimensions of real life, we know that no method is capable of reconstituting the totality. Our effortin this study was to look through the openings, catching a glimpse of the gaps and potential of the methods used to unveil existing mechanisms and contradictions. As stated by Nietzsche, we wanted “to stir up what was previously considered motionless, to fragment what was thought to be united; to show the heterogeneity of what was imagined in conformity with itself” [48].

## 6. Conclusions

In view of our findings, we demonstrated that structural issues are essential in the association between equity and efficiency and that intersectionality, along with its implications on health effectiveness, may be more comprehensively understood at the local level. Furthermore, the contradictions in the capital of the state of SP revealed that overcoming mortality in the first year of life does not guarantee living longer and better in this urban space, where wealth and vulnerability, opportunities and unemployment coexist.

The cost to society is extremely high, perverse and cruel, considering the chasm between the small average increments in Life Expectancy, which only a minority will be able to enjoy, and the precarious living and health conditions of those who sustain such *gains*. This happens exactly because the logic of capital has extended itself to all social relations and dominates all aspects of life.

These inequities are also reproduced inside the organizational arrangements of health actions and services in SP, as the expected equity was projected at the level of access to health and not the final results. This is an intersectoral aspect that should sensitize health managers, along with public local authorities in providing adequate local services and implementing public policies for improving living conditions, as well as tackling the existing social vulnerabilities and inequities, offering employment opportunities and adequate environmental conditions, in order to permit a thriving society. It cannot be stated, in any way, that the most efficient regions are the ones with the best living conditions, but that they are social spaces that use their resources in a more distributive way. Therefore, we can verify the importance of intersectional equity for achieving efficiency and effectiveness in local health systems, which is related to the choice (in time and space) of different political options of economic development models, radiating well-being comprehensively or concentrating it for only a privileged few.

## Figures and Tables

**Table 1:** Variables tested in the initial models by dimension and data sources.

Dimension/Variable	Variables	Data Source
**Physical/financial resources**	*Per capita* health expenditures derived from local tax revenues * % GDP destined to the service sector * % of health local revenues * Nurse density *Per capita* GDP % population receiving < ¼ MW % IPVS6 very high vulnerability Average *per capita* income	SES/SP SES/SP SES/SP CNES/DATASUS IBGE IBGE SEADE IBGE
**Health production**	% of hospital admissions sensitive to primary care * % overall vaccination coverage * Stroke hospitalization rate * % PHC coverage * % breast cancer screening % cervical cancer screening % newborns whose mothers had at least seven prenatal consultations Overall proportion of Caesarean sections	SIA/SIH PNI CIH SIAB SISMAMA SISCOLO SINASC SINASC (DATASUS)
**Environmental/intersectoral**	% vulnerable population dependent on the elderly * Income-ethnicity ratio (white/black and brown populations) % population without electricity access Theil index % of adolescent mothers % of water supply Aging rate Illiteracy rate Ratio of % of high level of education by gender and ethnicity (white men/black and brown women) Unemployment rate	IBGE IBGE IBGE PNUD IBGE IBGE IBGE IBGE IBGE IBGE

Note: CNES—National Register of Health Facilities; SIA—Ambulatory Information System; SIAB—Primary Care Information System; CIH—Hospital Information Communication; SINASC—Live Births Information System; SIH—Hospital Information System; SISCOLO and SISMAMA—Cervical and Breast Cancer Information Systems; PNI—National Immunization Program. IPVS—Sao Paulo Social Vulnerability Index/SEADE; UNDP: United Nations Development Program. * variables that did not remain in the final models.

**Table 2:** Final FE regression models for the dependent variables, related to dimensions and to the complete set models.

Dimension/Variable	LEB	IM
**Physical and Financial Resources**	% population receiving < ¼ MW (-) % IPVS6 very high vulnerability (-) Average *per capita* income (R^2^ = 71.22%; *p* < 0.0001)	% population receiving < ¼ MW % IPVS6 very high vulnerability Average *per capita* income (-) *Per capita* GDP (-) Nurse density (R^2^ = 71.35%; *p* < 0.0001)
**Health Production**	% Breast cancer screening (-) % Cervical cancer screening % newborns whose mothers had at least seven prenatal consultations (-) Overall proportion of Caesarean sections (R^2^ = 5.17%; *p* < 0.0001)	% Breast cancer screening % Cervical cancer screening (-) % newborns whose mothers had at least seven prenatal consultations Overall proportion of Caesarean sections (-) (R^2^ = 5.06%; *p* < 0.0001)
**Environment/intersectoral**	Theil Index (-) % adolescent mothers (-) % Water supply Aging rate Illiteracy rate (-) Ratio of % high level of education, gender and ethnicity (-) Unemployment rate (-) (R^2^ = 79.96%; *p* < 0.0001)	Theil index % children out of school % population without electricity access Aging rate (-) Ethnicity-income ratio (-) Ratio of % high level of education, gender and ethnicity Unemployment rate (R^2^ = 82.03%; *p* < 0.0001)
**Overall—all dimensions**	% population receiving < ¼ MW (-) % IPVS6 very high vulnerability (-) Average *per capita* income Theil Index (-) % adolescent mothers (-) Aging rate Illiteracy rate (-) Unemployment rate (-) % Water supply (R^2^ = 81.06%; *p* < 0.0001)	% population receiving < ¼ MW %IPVS6 very high vulnerability Ethnicity-income ratio (-) Theil Work Index % of children (0–5 years) out of school Aging rate (-) Illiteracy rate Unemployment rate (R^2^ = 81.87%; *p* < 0.0001)

Source: DATASUS; IBGE, SES/SP—SIOPS/MS and STN/MF (National Treasury Secretariat/Ministry of Finance); SEADE, PNUD/BRASIL and MPF (Federal Prosecution Ministry).

**Table 3:** Synthesis of health levels results, efficiency and contextual variables, according to the RRAS. Municipalities of the State of SP, 2000 and 2010.

RRAS	Name	Health Levels	Efficiency	Efficiency	Efficiency	Equity	Vulnerability	Education	Opportunities	Living Conditions
		Results	Synthesis	Fixed Effects	DEA	Variables	Variables	Variables	Variables	Variables
1	GSP—ABC	high	medium-high	medium-high	high	medium (I)	medium-high	high	low	high
2	GSP—Alto Tietê	low	Medium	high	medium-low	medium-high (I)	medium-high	medium-low	low	medium
3	GSP—Franco da Rocha	medium	medium-high	high	medium	High (I)	high	medium-high	low	medium
4	GSP—Mananciais	medium	medium-high	high	medium-high	medium-high (I)	high	medium	low	medium
5	GSP—Rota dos Bandeirantes	high	medium-high	medium-high	medium-high	medium-high (IIe)	medium-high	medium-high	low	high
6	GSP—SP	high	medium-low	low	medium	Low (IIwIeE)	medium-high	high	medium	high
7	Registro and Santos	medium-low	Medium	medium-high	medium-low	medium-low (I)	medium-high	medium-low	medium	low
8	Sorocaba	low	medium-low	medium	low	medium (Iw)	medium-high	low	medium	low
9	Bauru	medium	medium-low	low	medium-low	medium-high (E)	medium	medium-low	high	medium
10	Marília	low	medium-low	low	medium	Medium (E)	medium-low	medium	high	medium-low
11	Presidente Prudente	low	medium-high	medium	medium-high	medium-low (Iw)	medium-low	medium-low	medium-high	low
12	Araçatuba and SJ Rio Preto	medium-low	medium-low	low	medium	medium-high (E)	low	medium-low	high	medium-low
13	Ribeirão Preto, Araraquara, Barretos, and Franca	medium	medium-low	low	medium-low	Medium (EIe)	medium-low	medium-low	medium	medium-high
14	Piracicaba	high	Medium	medium	medium	medium-low (EIe)	medium-low	medium-high	medium	high
15	Campinas and S João Boa Vista	high	medium-high	medium	high	medium-low (EIe)	low	medium-high	high	medium-high
16	Campinas	medium	medium-low	medium	medium-low	medium-high (E)	medium-low	medium	medium-high	medium-low
17	Taubaté	medium	medium-high	medium-high	medium	medium-low (I)	medium-low	medium-high	medium	medium-low

Source: created by the authors, based on the performed regression models and the efficiency analysis (FE and DEA). Note: The above score results from the ranking among the RRAS (the top five with high scores and the lowest five with low scores; the remaining, medium). Equity variables derive from the distribution of general income (I), work (Iw) and ethnicity (Ie) and the distribution of education by gender and ethnicity (E), highlighting the worst regional distributions. Vulnerability variables refer to IPVS6 (high vulnerability) and the proportion of the population that receives < ¼ MW. Education variables derive from the literacy rate; % of non-adolescent mothers and % of children aged 0–5 years attending school; opportunities refer to employment and aging; the ones related to living conditions refer to average *per capita* income and adequate sanitation.

## Data Availability

The datasets (accessed on 19 June 2020) used during the current study are available from the corresponding author upon reasonable request. The consulted data sources are open to public access and can be reached at the following websites: IBGE—Brazilian Institute of Geography and Statistics, https://www.ibge.gov.br/estatisticas/sociais/populacao/9662-censo-demografico-2010.html?=&t=o-que-e; DATASUS—SUS Information Technology Department, https://datasus.saude.gov.br/informacoes-de-saude-tabnet/; SES/SP—Sao Paulo State Health Department, http://www.saude.sp.gov.br/ses/perfil/profissional-da-saude/informacoes-de-saude-/; SEADE—State System Foundation of Data Analysis, https://www.seade.gov.br/lista-produtos/; MPF—Federal Prosecution Ministry, http://combateacorrupcao.mpf.mp.br/ranking; SIOPS—Public Health Budget Information System, http://siops-asp.datasus.gov.br/cgi/siops/serhist/MUNICIPIO/indicadores.HTM; STN/MF—National Treasure Department/Ministry of Finance, https://www.gov.br/tesouronacional/pt-br; All data analyzed during this study are included in this published article (and its supplementary information files).

## Supplementary Materials

The following supporting information can be downloaded at: https://www.mdpi.com/article/10.3390/ijerph19052990/s1, Table S1: Results of the bivariate analysis, fixed effects model, for the variables life expectancy and infant mortality: resources, health and intersectoral dimensions. Sao Paulo Municipalities, 2000 and 2010. Description: All variables tested initially in the fixed effects bivariate analysis can be consulted in these tables, according to the physical and financial resources; the health production and the intersectoral dimension. Note that only some variables were directed to the initial multivariate models. Table S2: Results of the bivariate analysis, fixed effects model, for the variables life expectancy and infant mortality—intersectoral dimension. Sao Paulo Municipalities, 2000 and 2010. Table S3: Results of the bivariate analysis, fixed effects model, for the variables life expectancy and infant mortality—health production dimension. Sao Paulo Municipalities, 2000 and 2010. Figure S1: Potential years gained in life expectancy at birth and reduction in infant mortality rates, regionalized healthcare networks and health regions: comparison between methods (FE × DEA). Description: This file depicts the number of potential years gained in life expectancy at birth and the potential reduction in infant mortality rates, calculated with both techniques used, permitting comparisons between municipalities, within health regions.

## Abbreviations

FE	Fixed Effects
DEA	Data Envelopment Analysis
LEB	life expectancy at birth
IM	infant mortality
RRAS	regionalized healthcare networks
HR	health regions
HDI	Human Development Index
IPVS	Sao Paulo Social Vulnerability Index
IPRS	Sao Paulo Social Responsibility Index
MPF	Federal Prosecution Ministry
MW	minimum wage
PHC	Primary Health Care
