# Supplementary material for: Efficiency Analysis in Brazil’s Sao Paulo State Local Unified Health System (SUS): From Gender-Ethnicity-Power Inequities to the Dissolution of Health Effectiveness
Source: Int J Environ Res Public Health. 2022 Mar 4;19(5):2990. doi: 10.3390/ijerph19052990 (PMC8910277; doi:10.3390/ijerph19052990)

Supplementary Materials

Table S1 - Results of the bivariate analysis, fixed effects model, for the variables life expectancy and infant mortality, financial and physical resources dimension. Sao Paulo Municipalities, 2000 and 2010.

Resources dimension variables	Life Expectancy at birth	Infant Mortality
Population IBGE	1,53 e-05 (<0,001)	-2,42 e-05 (<P0,001)
Per capita GDP IBGE	1,01e-05 (0,088)	-1,67e-05 (0,099)
GDP IBGE	1,85 e-08 (0,263)	-2,71 e-08 (0,338)
% state GDP SES	-0,0005 (0,434)	0,0006 (0,602)
Current per capita health expenditure SES	0,0010 (0,002)	0,0010 (0,002)
Health expenditures as % GDP SES	13,6442 (0,184)	-21,2698 (0,227)
Wealth (IPRS) SEADE	-0,0657 (0,237)	0,1321 (0,165)
Longevity (IPRS) SEADE	0,1090 (0,240)	-0,0322 (0,246)
IPVS 1 extremely low SEADE	-1,0161 (0,876)	1,8841 (0,866)
IPVS 2 very low	1,0918 (0,053)	-1,5946 (0,100)
IPVS 3 low	0,9769 (0,326)	-1,8650 (0,274)
IPVS 4 medium	1,2963 (<0,001)	-2,2000 (<0,001)
IPVS 5 high	-0,8527 (<0,001)	1,4385 (<0,001)
IPVS 6 very high	-2,7525 (<0,001)	4,8463 (<0,001)
Density of physicians CNES	0,2684 (0,216)	-0,4694 (0,207)
Density of nurses	0,2051 (0,004)	-0,3232 (0,009)
Densidade de CT	-0,0006 (0,790)	0,0004 (0,913)
Densidade de MR	1,99 e-05 (0,955)	1,2 e-05 (0,984)
Densidade of Health Centers	0,0407 (0,780)	-0,0475 (0,850)
Density of beds SUS CNES	-0,2107 (0,300)	0,4170 (0,242)
Own health revenues SES	0,0642 (0,007)	-0,1087 (0,008)
Primary care % SES	-0,0100 (0,167)	0,0158 (0,205)
Farming (%) IBGE	-2,5709 (0,062)	4,1117 (0,081)
Industries (%)	-0,4144 (0,762)	0,4698 (0,842)
Services (%)	3,6693 (0,034)	-5,5380 (0,062)
Taxes (%)	-1,2129 (0,750)	2,0612 (0,752)
Per capita own health revenues SES	0,0014 (0,001)	-0,0024 (0,002)
Population earning <1/2 MW (%) IBGE<br>In the black and brown population	-0,1059 (<0,001)<br>-0,0193 (<0,001)	0,1805 (<0,001)<br>0,0314 (<0,001)
Population earning <1/4 MW (%)<br>In the black and brown population	-0,2675 (<0,001)<br>-0,1765 (<0,001)	0,4625 (<0,001)<br>0,3028 (<0,001)
Average income IBGE<br>In the black and brown population	0,0145 (<0,001)<br>0,0111 (<0,001)	-0,0244 (<0,001)<br>-0,0190 (<0,001)

Source: DATASUS; IBGE, SES/SP - SIOPS/MS and STN/MF (National Treasure Secretary/ Ministry of Finance); SEADE. MW – minimum wage; IPRS – Social Responsibility index (SP); IPVS – Social Vulnerability index (SP).

Notes: IBGE –Brazilian Institute of Geography and Statistics; DATASUS – SUS Information Technology Department; SES/SP - Sao Paulo State Health Department; SEADE – State System Foundation of Data Analysis

Table S2 - Results of the bivariate analysis, fixed effects model, for the variables life expectancy and infant mortality - intersectoral dimension. Sao Paulo Municipalities, 2000 and 2010.

Intersectoral dimension variables	Life Expectancy at birth	Infant Mortality
**Gini index IBGE**<br>**Income inequality - quintiles**<br>**Theil L index UNDP**<br>**Theil work income index**	-5,5423 (<0,001)<br>-0,0911 (<0,001)<br>-14,1522 (<0,001)<br>-12,5916 (<0,001)	9,5573 (0,001)<br>0,1585 (<0,001)<br>24,3542 (<0,001)<br>21,4014 (<0,001)
**% teenage motherhood IBGE**<br>**% head of household mothers low education level and dependent offspring**<br>**% children out of school (0-5 years)**<br>**% vulnerable populations low Education level**<br>**% population dependent upon the elderly**<br>**% vulnerable populations without electricity**<br>**% vulnerable poor population**<br>**Per capita income in poor vulnerable population**	-0,3393 (<0,001)<br>0,2582 (<0,001)<br>-0,0897 (<0,001)<br>-0,1137 (<0,001)<br>-0,1849 (<0,001)<br>-1,2212 (<0,001)<br>-0,4587 (<0,001)<br>-0,1409 (<0,001)<br>0,1149 (<0,001)	0,5799 (<0,001)<br>-0,4428 (<0,001)<br>0,1525 (<0,001)<br>0,1931 (<0,001)<br>0,3184 (<0,001)<br>2,0979 (<0,001)<br>0,8345 (<0,001)<br>0,2410 (<0,001)<br>-0,1974 (<0,001)
**Average Income ethnicity ratio (white/black and brown population) IBGE**<br>**Population earning <1/2 MW ethnicity ratio**<br>**Population earning<1/4 MW ethnicity ratio**	-4,0293 (<0,001)<br>-1,7657 (<0,001)<br>0,8524 (<0,001)	6,8769 (<0,001)<br>3,0025 (<0,001)<br>-1,4380 (<0,001)
**Average income gender ratio (M/F) IBGE**	-0,3791 (0,530)	0,6593 (0,524)
**HDIM (municipal) PNUD**<br>**HDIM Income**	32,8998 (<0,001)<br>52,1460 (<0,001)	-56,2390 (<0,001)<br>-88,8587 (<0,001)
**HDIM –Education**<br>**HDIM Longevity**	18,4177(<0,001)<br>59,9864 (<0,001)	-31,5047 (<0,001)<br>-102,3235 (<0,001)
**Dependency ratio IBGE**<br>**Aging rate**	-0,3457 (<0,001)<br>1,2903 (<0,001)	0,5918 (<0,001)<br>-2,0907 (<0,001)
**Black and brown population (%) IBGE**	25,6972 (<0,001)	-43,7407 (<0,001)
**At least one deficiency (% population) IBGE**	5,1235 (<0,001)	-8,3994 (<0,001)

**Education level (iprs – adequação port/mat no 5º e 9º ano EF, atend 0-3 anos e distorção idade-série EM) SEADE**	0,0422 (<0,001)	-0,0692 (<0,001)
**Water quality SES**	0,0029 (0,853)	-0,0073 (0,785)
**Basic sanitation IBGE**		
**Water supply**	0,3440 (<0,001)	-0,5893 (<0,001)
**Sanitary facilities**	0,1606 (<0,001)	-0,2744 (<0,001)
**Waste collection/disposal**	0,2082 (<0,001)	-0,3568 (<0,001)
**Higher education (%) IBGE**	0,2071 (<0,001)	-0,3528 (<0,001)
**Female**	0,2020 (<0,001)	-0,3441 (<0,001)
**Male**	0,2069 (<0,001)	-0,3524 (<0,001)
**Black and brown pop**	0,1642 (<0,001)	-0,2791 (<0,001)
**White**	0,2010 (<0,001)	-0,3425 (<0,001)
**White male**	0,1995 (<0,001)	-0,3397 (<0,001)
**Black/brown female**	0,1566 (<0,001)	-0,2659 (<0,001)
**Gender and ethnicity inequity ratio**	-3,4405 (<0,001)	5,8159 (<0,001)
**Illiteracy rate IBGE**	-0,7352 (<0,001)	1,2537 (<0,001)
**Unemployment rate IBGE**	-0,3768 (<0,001)	0,6386 (<0,001)
**In the black and brown population**	-0,2865 (<0,001)	0,4832 (<0,001)
**Transparency level (MPF)**	0,4783 (0,373)	-0,0677 (0,467)

Source: DATASUS; IBGE, SIOPS/MS and STN/MF (National Treasure Secretariat/ Ministry of Finance); SEADE, PNUD/BRASIL e MPF (Federal Prosecution Ministry). PNUD - UNDP United Nations Development Programme.

Notes: IBGE –Brazilian Institute of Geography and Statistics; DATASUS – SUS Information Technology Department; SES/SP - Sao Paulo State Health Department; SEADE – State System Foundation of Data Analysis

Table S3 - Results of the bivariate analysis, fixed effects model, for the variables life expectancy and infant mortality – health production dimension. Sao Paulo Municipalities, 2000 and 2010.

Health dimension variables	Life Expectancy at birth	Infant Mortality
**Supplementary health coverage ANS**	0,0186 (0,256)	-0,0329 (0,243)
**PHC coverage SIAB**	0,0026 (0,142)	-0,0044 (0,142)
**PHC sensitive hospital admissions SIA/SIH**	-0,0181 (0,207)	0,0287 (0,249)
**Breast cancer screening SISMAMA**	-1,3169 (0,014)	2,3626 (0,010)
**Cervical cancer screening SISCOLO**	0,3709 (0,020)	-0,6187 (0,024)
**Newborn whose mothers attended at least seven prenatal consultations SINASC**	-0,0312 (0,008)	0,0526 (0,010)
**PHC medical consultations SIAB**	-0,0214 (0,364)	0,0363 (0,369)
**Hospital admissions SUS CIH**	-0,0362 (0,688)	0,0450 (0,771)

**Vaccine coverage PNI**	0,0070 (0,171)	-0,0111 (0,207)
**TB treatment SINAN**	-0,0044 (0,481)	0,0096 (0,363)
**Vaccination general index (%) PNI**	0,0036 (0,140)	-0,0053 (0,204)
**CVA hospital admissions CIH**	-0,0023 (0,114)	0,0036 (0,155)
**% cesarean sections SINASC**	0,0286 (<0,001)	-0,0482 (<0,001)
**Hip fracture hospital admissions CIH**	-0,0013 (0,600)	0,0016 (0,696)

Source: DATASUS; IBGE, SES/SP and SEADE.

Notes: CNES - National Registry of Health Facilities; SINASC – Live Births Information System; SINAN – Notifiable Diseases Information System; SIA – Ambulatory Information System; SIAB – PHC Information System; CIH – Hospital Information Communication; SIM – Mortality Information System; SISCOLO and SISMAMA – Cervical and breast Cancer Information Systems; PNI – National Immunization Programme. IBGE –Brazilian Institute of Geography and Statistics; DATASUS – SUS Information Technology Department; SES/SP - Sao Paulo State Health Department; SEADE – State System Foundation of Data Analysis

Supplementary Figure S1 Potential years gained in life expectancy at birth and reduction in infant mortality rates, regionalized healthcare networks and health regions: comparison between methods (FE × DEA).

Graph S1.1 – Potential years of life gained for ABC/Grande São Paulo (RRAS1), FE x DEA.

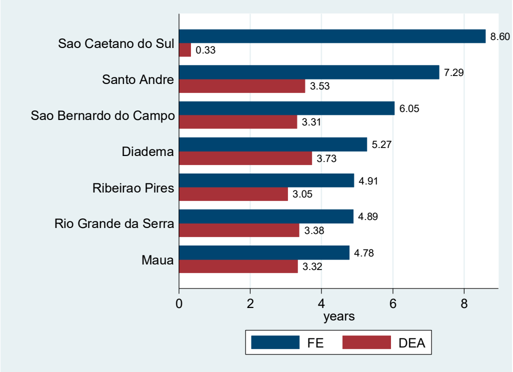

Graph S2.1 Potential reduction in infant mortality (IM) rates for ABC/Grande São Paulo (RRAS1), FE x DEA

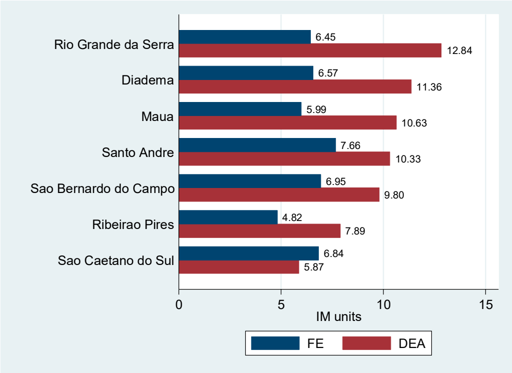

Graph S1.2 - Potential years of life gained for Alto Tietê (RRAS2), FE x DEA.

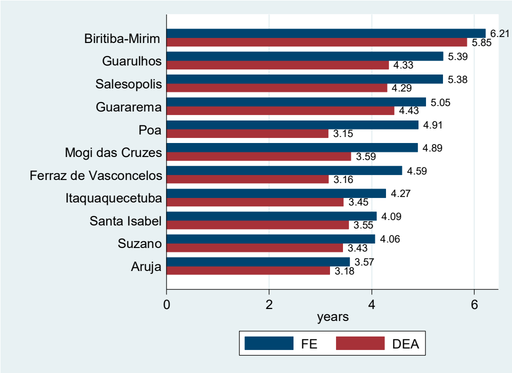

Graph S2.2- Potential reduction in IM rates for Alto Tietê (RRAS2), FE x DEA

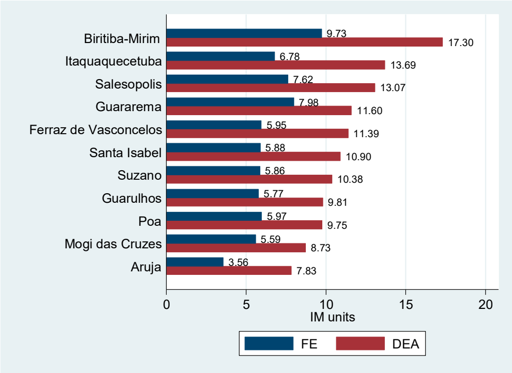

Graph S1.3 - Potential years of life gained for Franco da Rocha (RRAS3), FE x DEA.

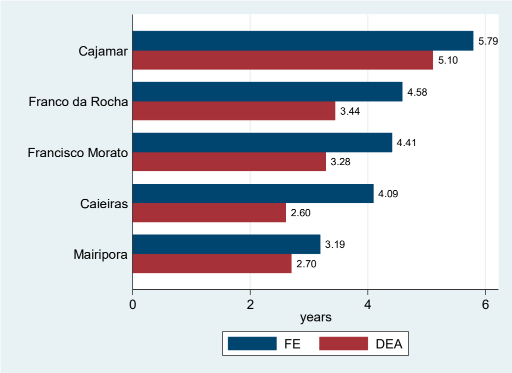

Graph S2.3- Potential reduction in IM rates for Franco da Rocha (RRAS3), FE x DEA

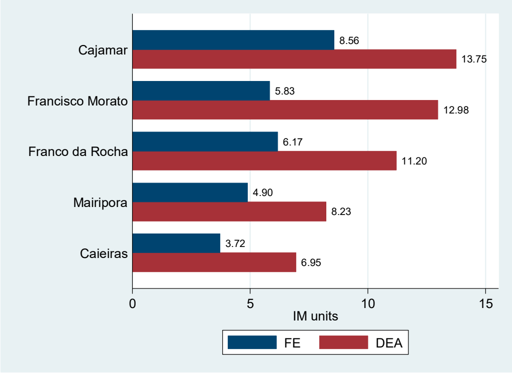

Graph S1.4 - Potential years of life gained for Mananciais (RRAS4), FE x DEA.

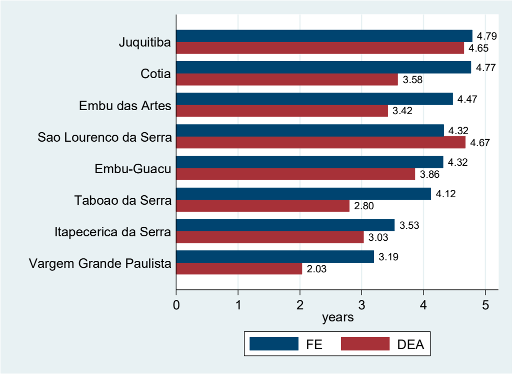

Graph S2.4- Potential reduction in IM rates for Mananciais (RRAS4), FE x DEA

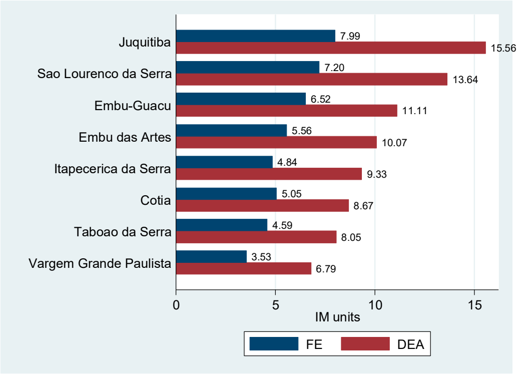

Graph S1.5 - Potential years of life gained for Rota dos Bandeirantes (RRAS5), FE x DEA.

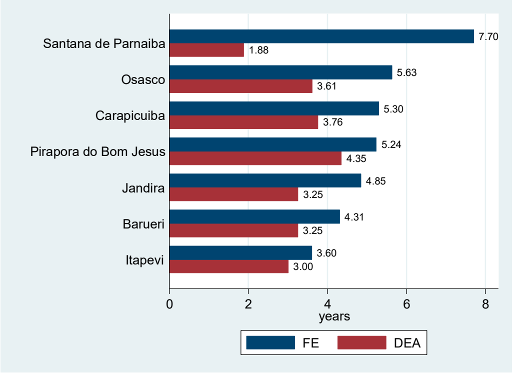

Graph S2.5- Potential reduction in IM rates for Rota dos Bandeirantes (RRAS5), FE x DEA

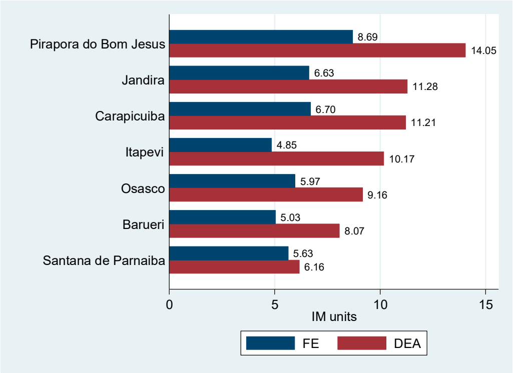

Graph S1.6 e S2.6 - Potential years of life gained and reduction in IM rates for São Paulo (RRAS6), FE x DEA.

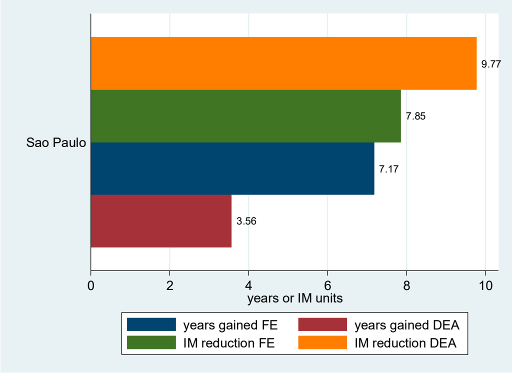

Graph S1.7 - Potential years of life gained for Registro and Baixada Santista (RRAS7), FE x DEA.

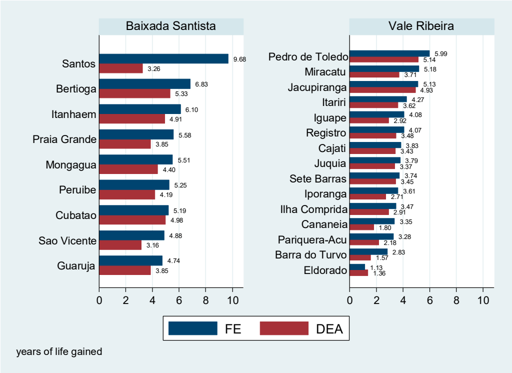

Graph S2.7- Potential reduction in IM rates for Registro and Baixada Santista (RRAS7), FE x DEA

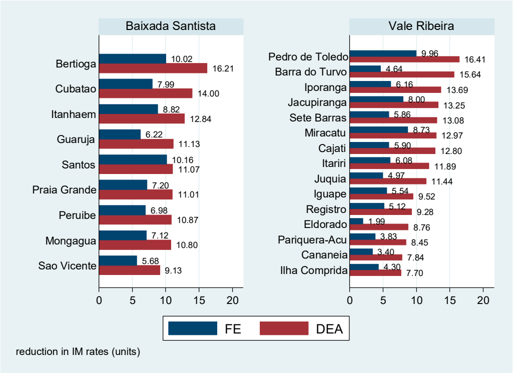

Graph S1.8.1 - Potential years of life gained for Itapetininga (RRAS8), FE x DEA.

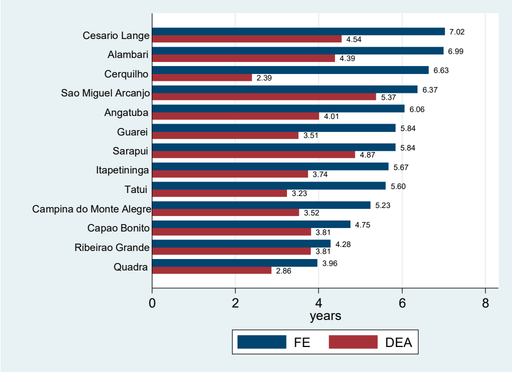

Graph S2.8.1- Potential reduction in IM rates for Itapetininga (RRAS8), FE x DEA

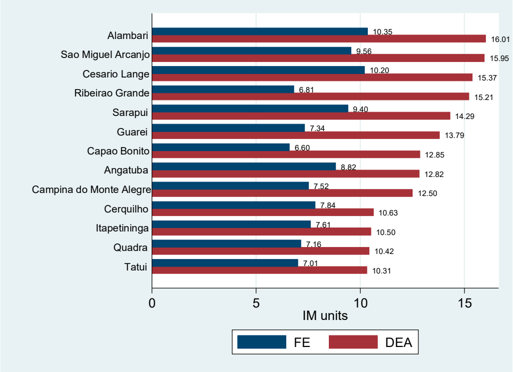

Graph S1.8.2 - Potential years of life gained for Itapeva (RRAS8), FE x DEA.

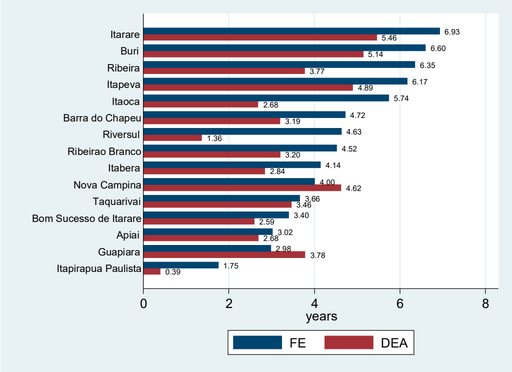

Graph S2.8.2- Potential reduction in IM rates for Itapeva (RRAS8), FE x DEA

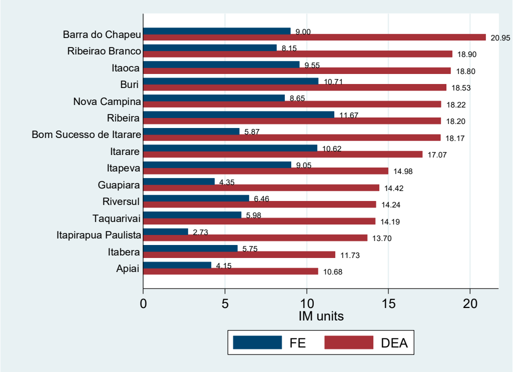

Graph S1.8.3 - Potential years of life gained for Sorocaba (RRAS8), FE x DEA.

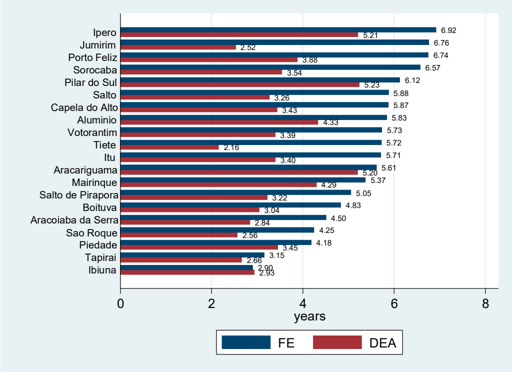

Graph S2.8.3- Potential reduction in IM rates for Sorocaba (RRAS8), FE x DEA

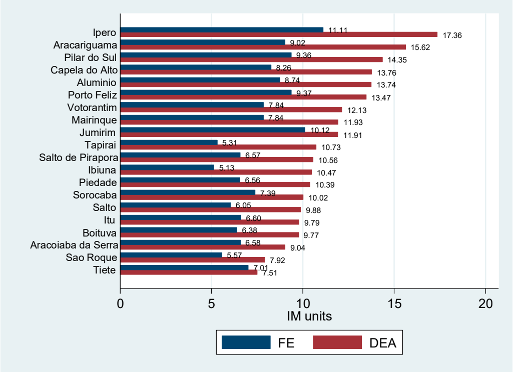

Graph S1.9.1 - Potential years of life gained for Bauru (RRAS9), FE x DEA.

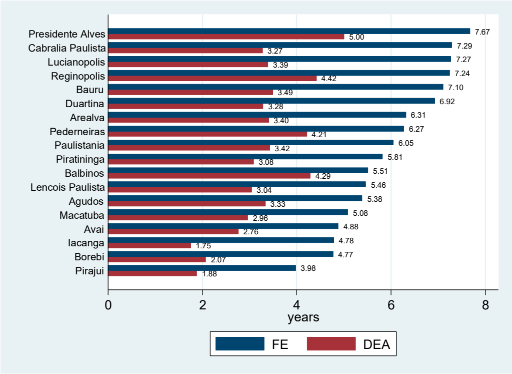

Graph S2.9.1- Potential reduction in IM rates for Bauru (RRAS9), FE x DEA

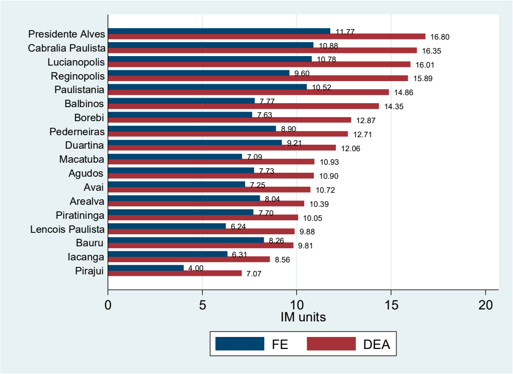

Graph S1.9.2 - Potential years of life gained for Jaú (RRAS9), FE x DEA.

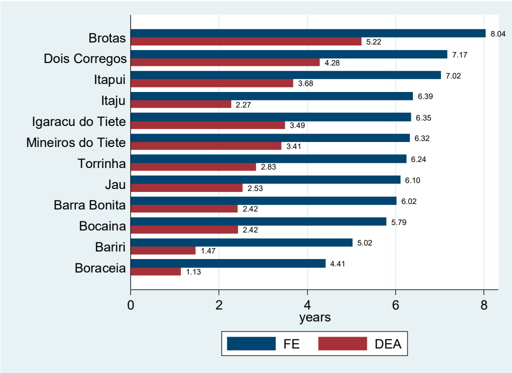

Graph S2.9.2- Potential reduction in IM rates for Jaú (RRAS9), FE x DEA

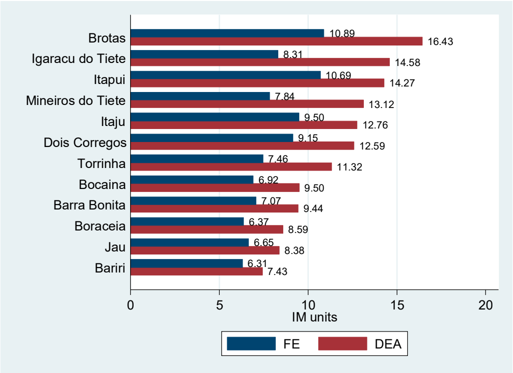

Graph S1.9.3 - Potential years of life gained for Jurumim (RRAS9), FE x DEA.

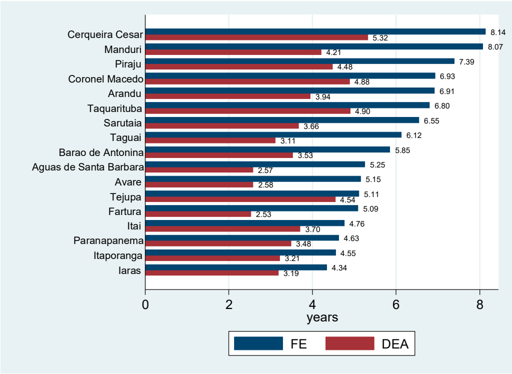

Graph S2.9.3- Potential reduction in IM rates for Jurumim (RRAS9), FE x DEA

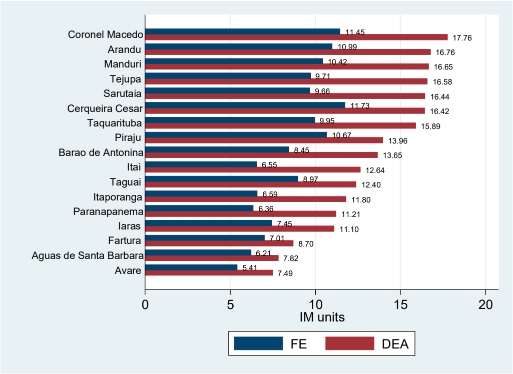

Graph S1.9.4 - Potential years of life gained for Lins (RRAS9), FE x DEA.

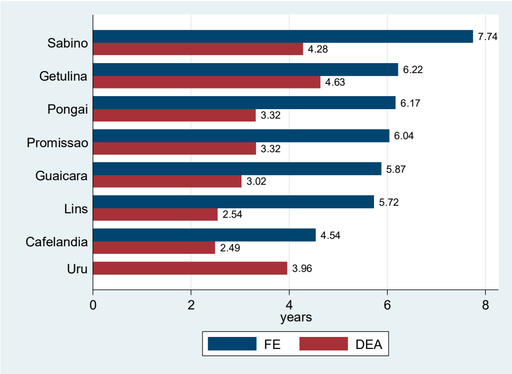

Graph S2.9.4- Potential reduction in IM rates for Lins (RRAS9), FE x DEA

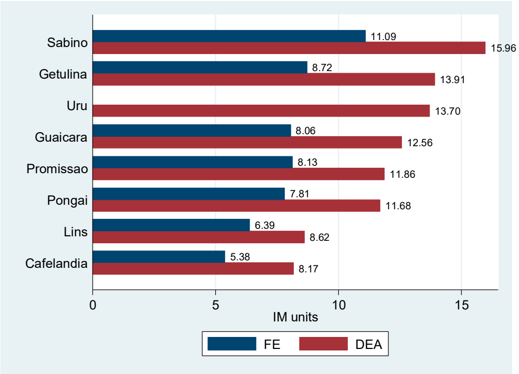

Graph S1.9.5 Potential years of life gained for Polo Cuesta (RRAS9), FE x DEA.

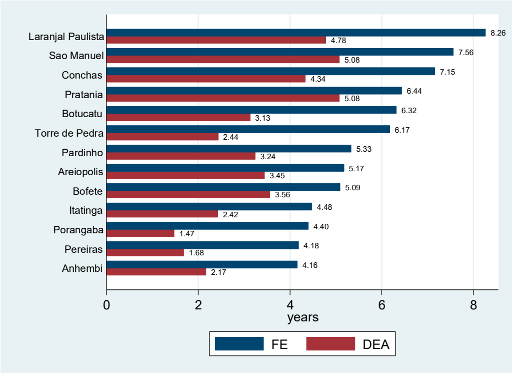

Graph S2.9.5- Potential reduction in IM rates for Polo Cuesta (RRAS9), FE x DEA

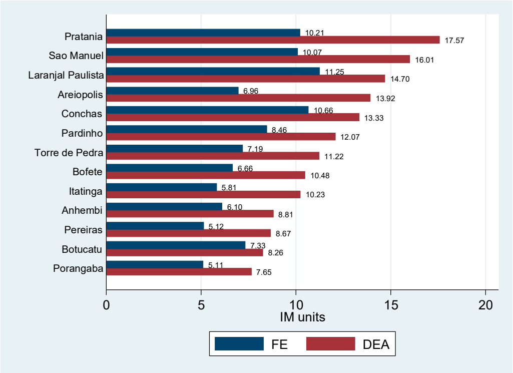

Graph S1.10.1 - Potential years of life gained for Adamantina (RRAS10), FE x DEA.

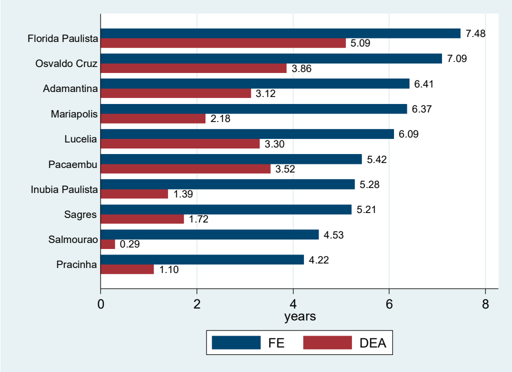

Graph S2.10.1- Potential reduction in IM rates for Adamantina (RRAS10), FE x DEA

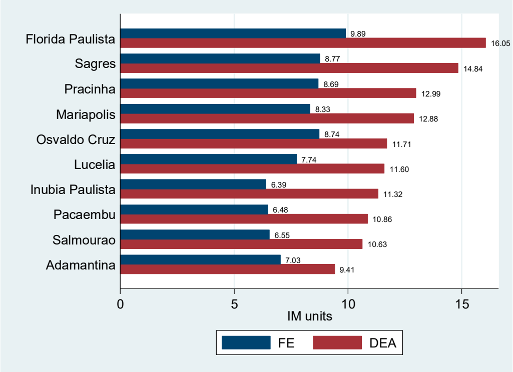

Graph S1.10.2 Potential years of life gained for Assis (RRAS10), FF x DEA.

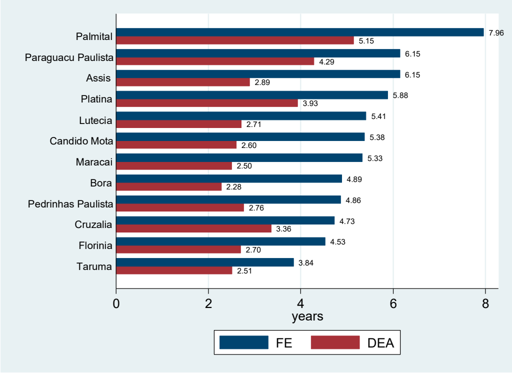

Graph S2.10.2- Potential reduction in IM rates for Assis (RRAS10), FE x DEA

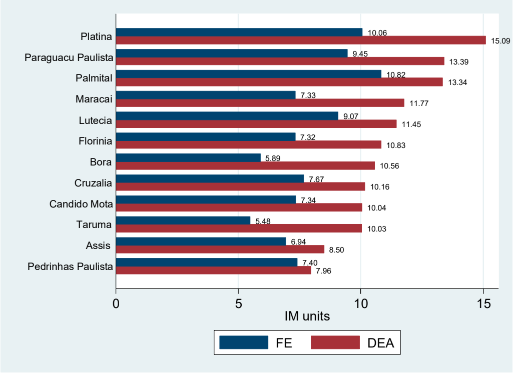

Graph S1.10.3 - Potential years of life gained for Ourinhos (RRAS10), FE x DEA.

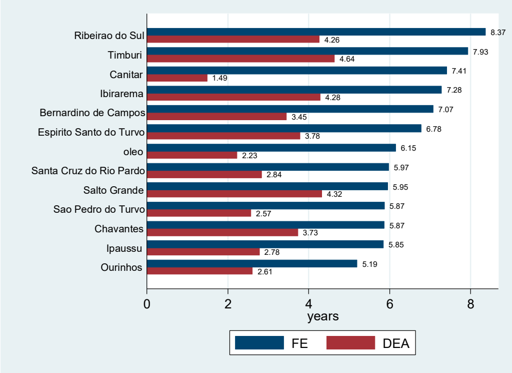

Graph S2.10.3- Potential reduction in IM rates for Ourinhos (RRAS10), FE x DEA

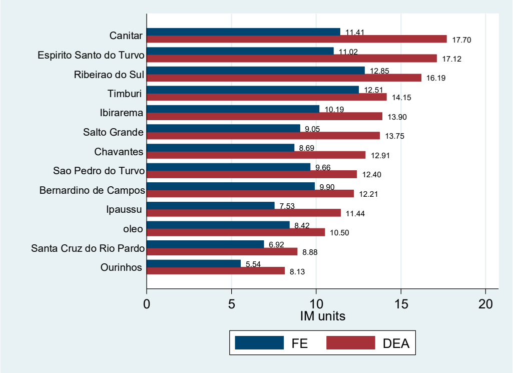

Graph S1.10.4 - Potential years of life gained for Marília (RRAS10), FE x DEA.

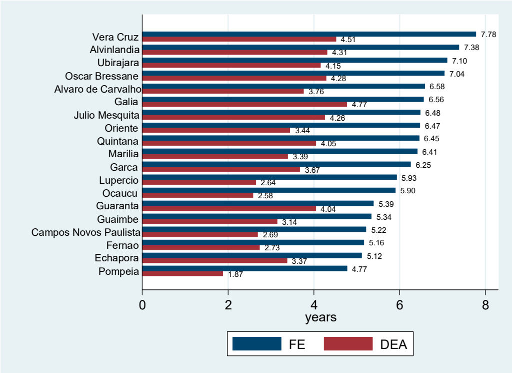

Graph S2.10.4- Potential reduction in IM rates for Marília (RRAS10), FE x DEA

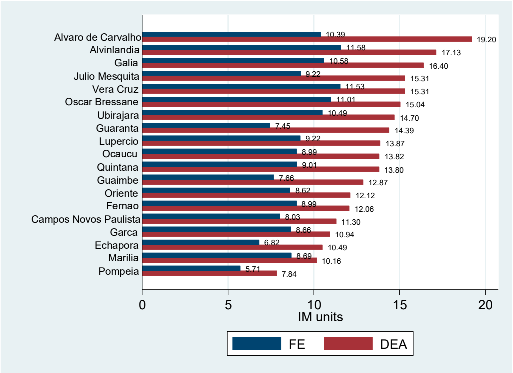

Graph S1.10.5 - Potential years of life gained for Tupã (RRAS10), FE x DEA.

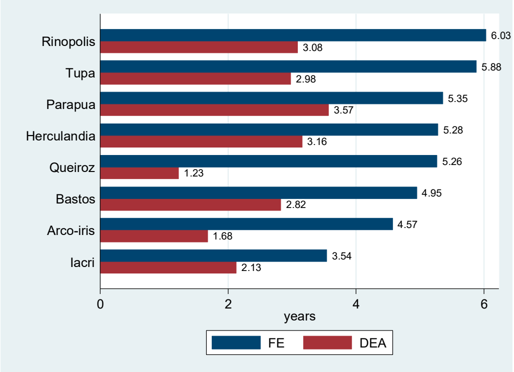

Graph S2.10.5- Potential reduction in IM rates for Tupã (RRAS10), FE x DEA

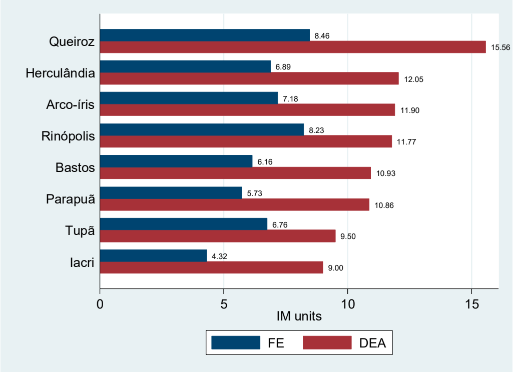

Graph S1.11.1 - Potential years of life gained for Alta Paulista (RRAS11), FE x DEA.

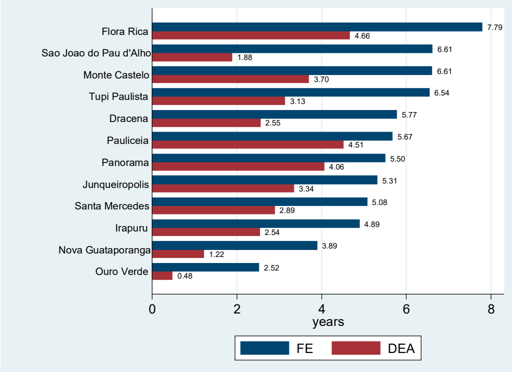

Graph S2.11.1- Potential reduction in IM rates for Alta Paulista (RRAS11), FE x DEA

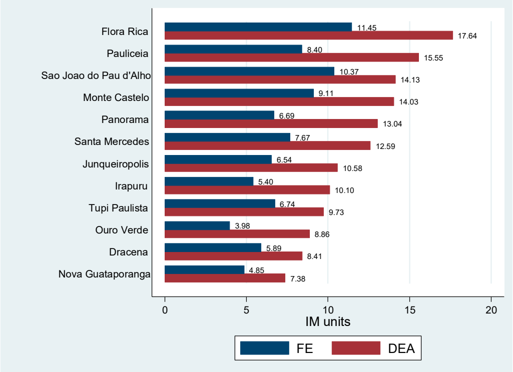

Graph S1.11.2 - Potential years of life gained for Alta Sorocabana (RRAS11), FE x DEA.

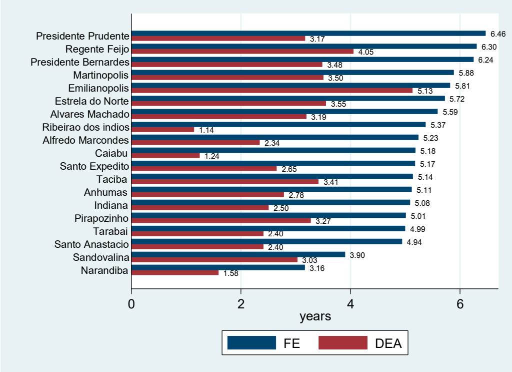

Graph S2.11.2- Potential reduction in IM rates for Alta Sorocabana (RRAS11), FE x DEA

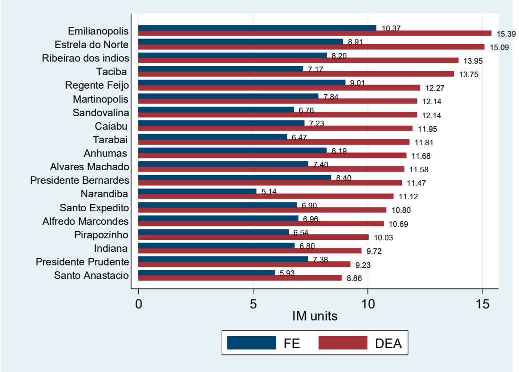

Graph S1.11.3 - Potential years of life gained for Alto Capivari (RRAS11), FE x DEA.

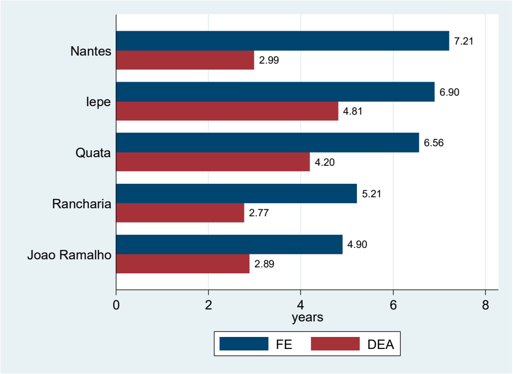

Graph S1.11.4 - Potential years of life gained for Extremo Oeste Paulista (RRAS11), FE x DEA.

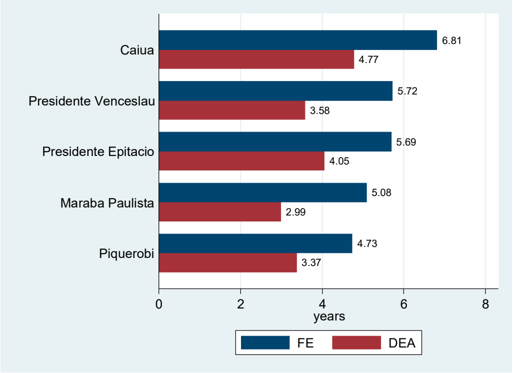

Graph S1.11.5 - Potential years of life gained for Pontal do Paranapanema (RRAS11), FE x DEA.

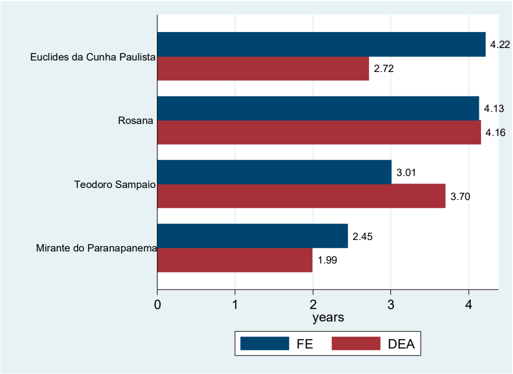

Graph S1.12.1 - Potential years of life gained for Catanduva (RRAS12), FE x DEA.

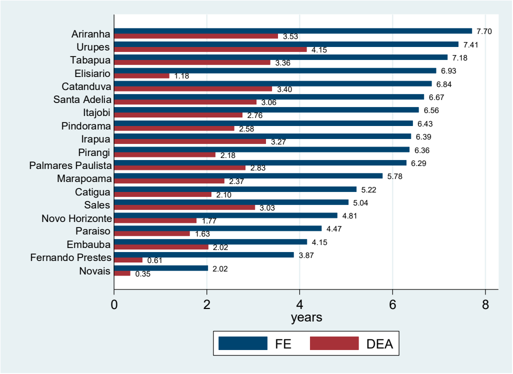

Graph S2.11.3- Potential reduction in IM rates for Alto Capivari (RRAS11), FE x DEA

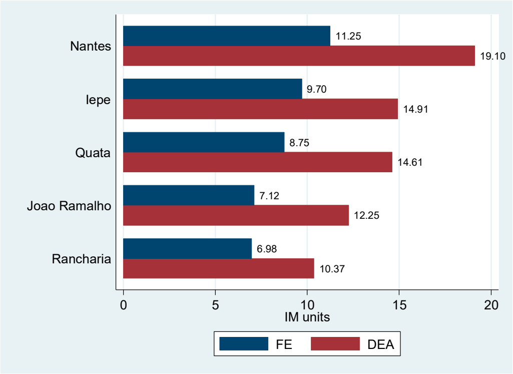

Graph S2.11.4- Potential reduction in IM rates for Extremo Oeste Paulista (RRAS11), FE x DEA

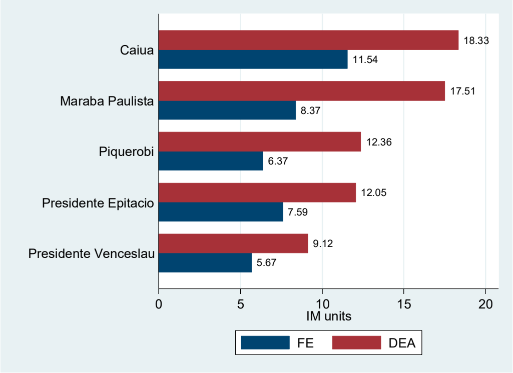

Graph S2.11.5- Potential reduction in IM rates for Pontal do Paranapanema (RRAS11), FE x DEA

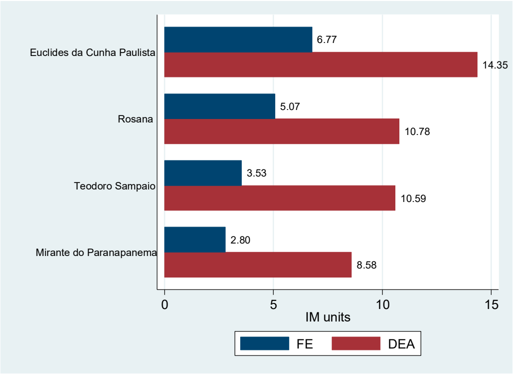

Graph S2.12.1- Potential reduction in IM rates for Catanduva (RRAS12), FE x DEA

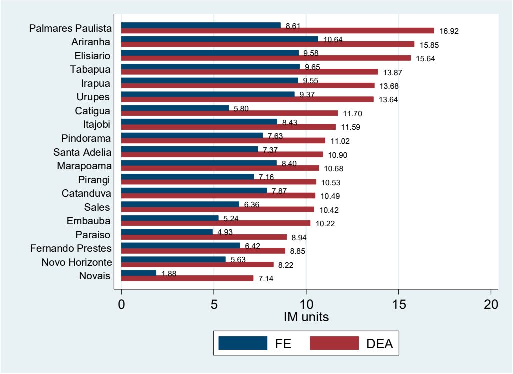

Graph S1.12.2 - Potential years of life gained for Central Araçatuba (RRAS12), FE x DEA.

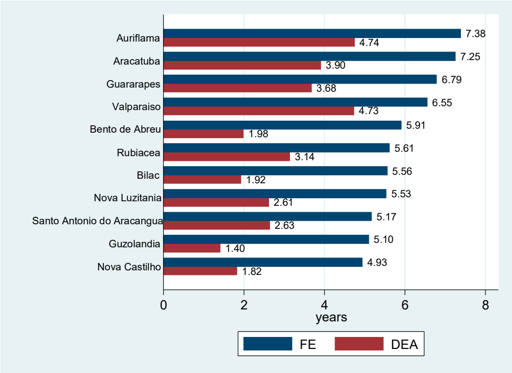

Graph S2.12.2- Potential reduction in IM rates for Central Araçatuba (RRAS12), FE x DEA

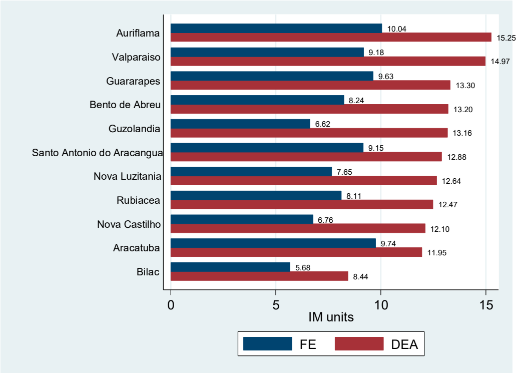

Graph S1.12.3 - Potential years of life gained for Consórcio Araçatuba (RRAS12), FE x DEA.

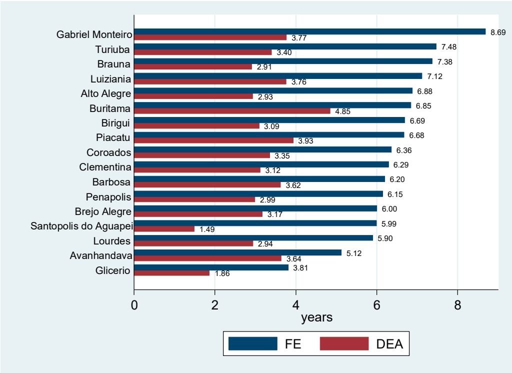

Graph S2.12.3- Potential reduction in IM rates for Consórcio Araçatuba (RRAS12), FE x DEA

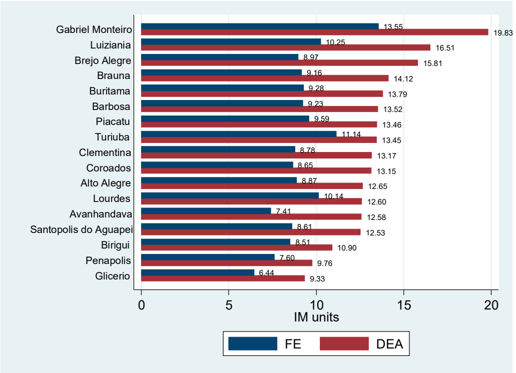

Graph S1.12.4 - Potential years of life gained for Fernandópolis (RRAS12), FE x DEA.

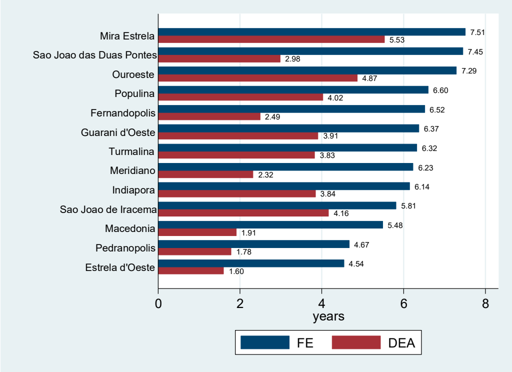

Graph S2.12.4- Potential reduction in IM rates for Fernandópolis (RRAS12), FE x DEA

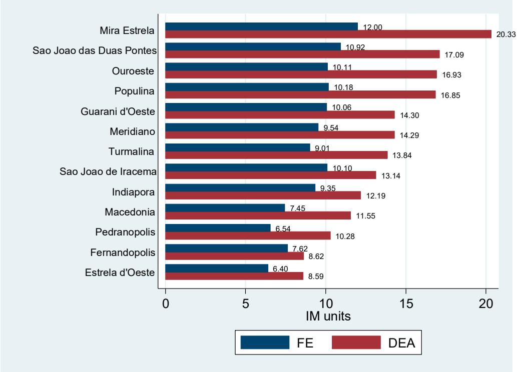

Graph S1.12.5 - Potential years of life gained for Jales (RRAS12), FE x DEA.

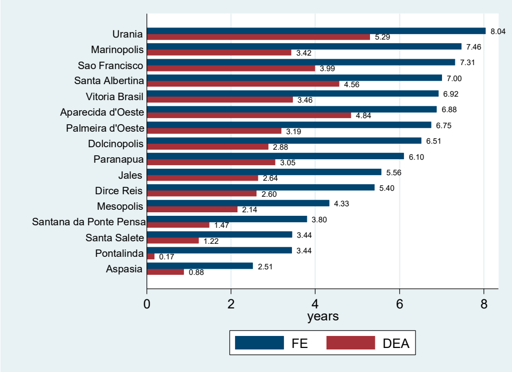

Graph S2.12.5- Potential reduction in IM rates for Jales (RRAS12), FE x DEA

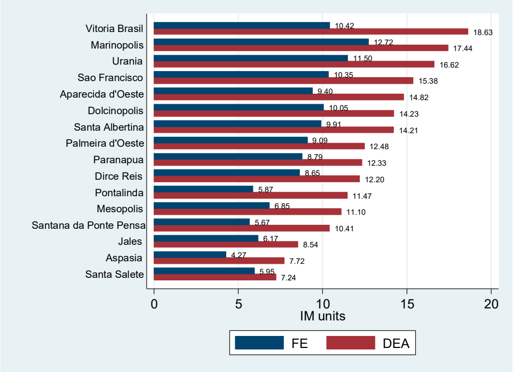

Graph S1.12.6 - Potential years of life gained for José Bonifácio (RRAS12), FE x DEA.

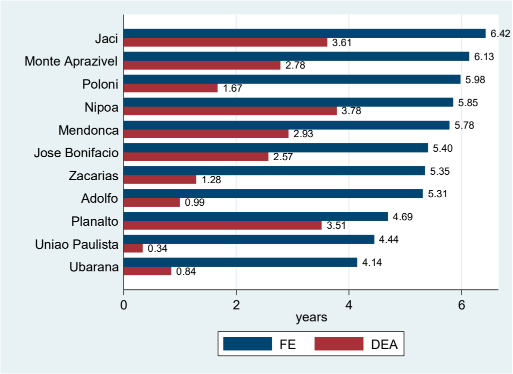

Graph S2.12.6- Potential reduction in IM rates for José Bonifácio (RRAS12), FE x DEA

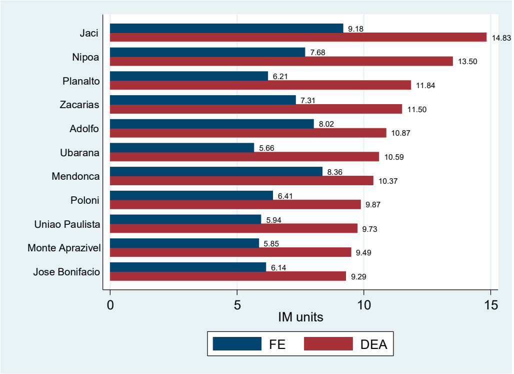

Graph S1.12.7 - Potential years of life gained for Lagos Araçatuba (RRAS12), FE x DEA.

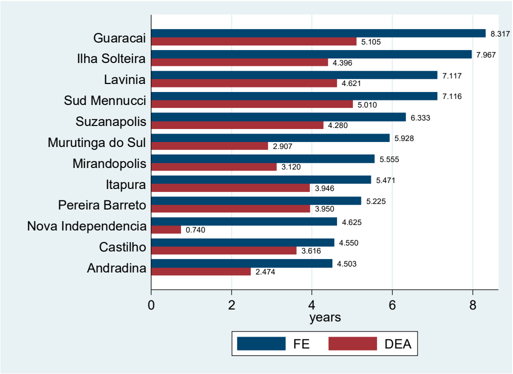

Graph S2.12.7- Potential reduction in IM rates for Lagos Araçatuba (RRAS12), FE x DEA

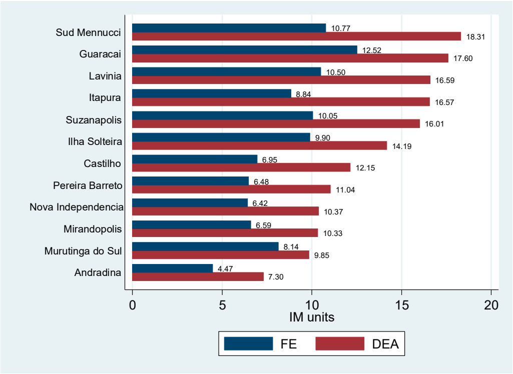

Graph S1.12.8 - Potential years of life gained for São José do Rio Preto (RRAS12), FE x DEA.

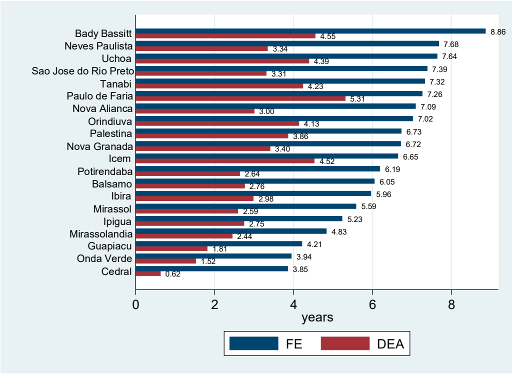

Graph S2.12.8- Potential reduction in IM rates for São José do Rio Preto (RAS12), FE x DEA

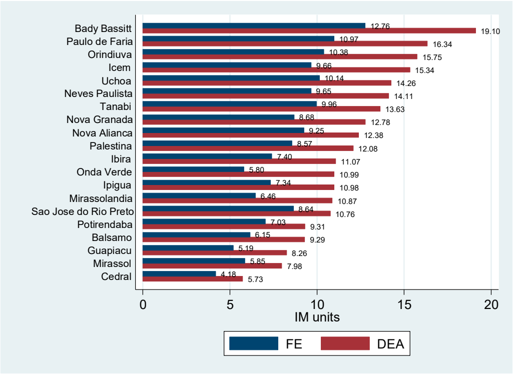

Graph S1.12.9 - Potential years of life gained for Santa Fé do Sul (RRAS12), FE x DEA.

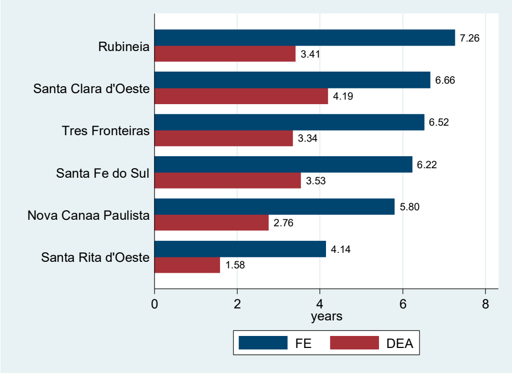

Graph S2.12.9- Potential reduction in IM rates for Santa Fé do Sul (RAS12), FE x DEA

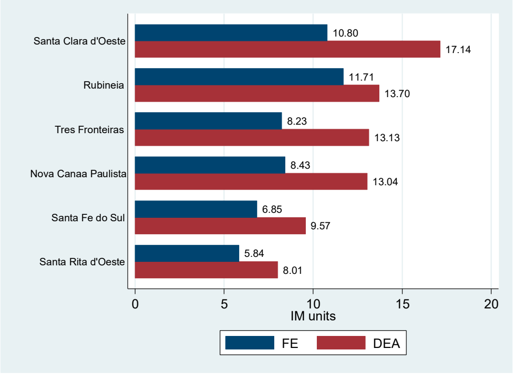

Graph S1.12.10 - Potential years of life gained for Votuporanga (RRAS12), FE x DEA.

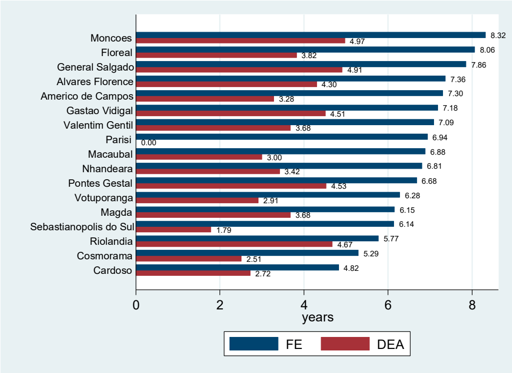

Graph S2.12.10- Potential reduction in IM rates for Votuporanga (RAS12), FE x DEA

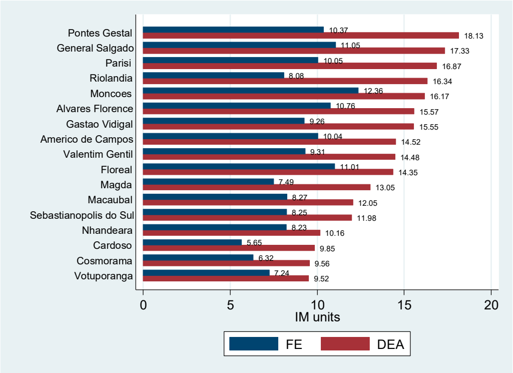

Graph S1.13.1 - Potential years of life gained for Alta Anhanguera (RRAS13), FE x DEA.

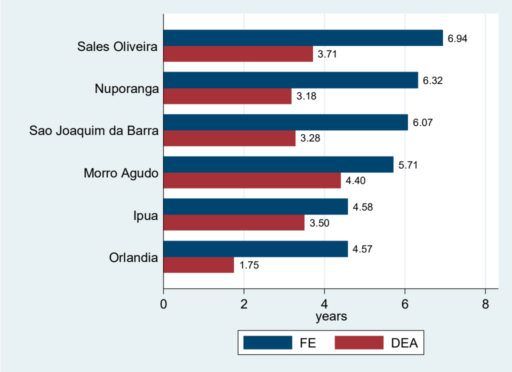

Graph S2.13.1- Potential reduction in IM rates for Alta Anhanguera (RAS13), FE x DEA

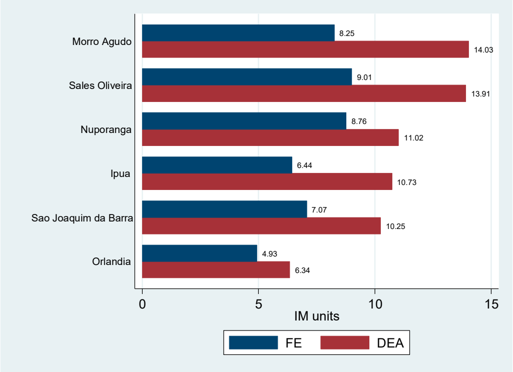

Graph S1.13.2 - Potential years of life gained for Alta Mogiana (RRAS13), FE x DEA.

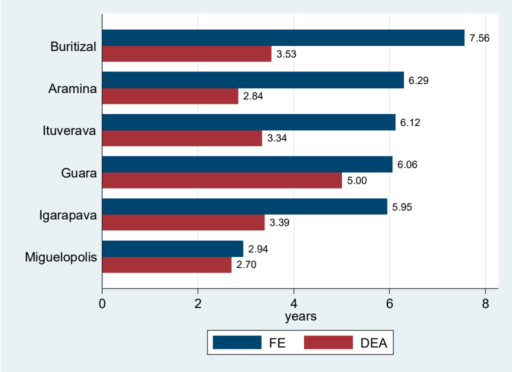

Graph S2.13.2- Potential reduction in IM rates for Alta Mogiana (RAS13), FE x DEA

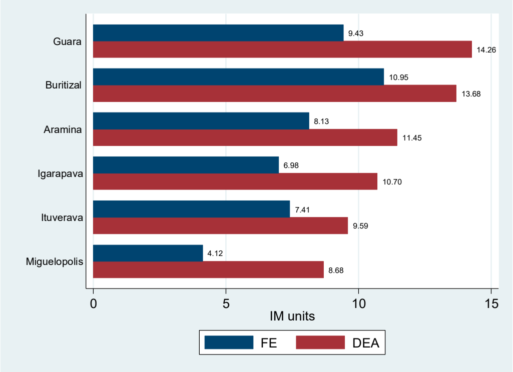

Graph S1.13.3 - Potential years of life gained for Aquífero Guarani (RRAS13), FE x DEA.

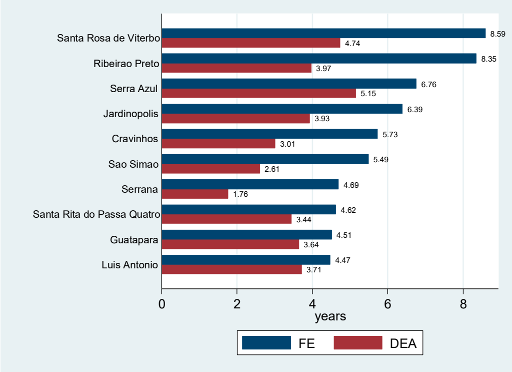

Graph S2.13.3- Potential reduction in IM rates for Aquífero Guarani (RAS13), FE x DEA

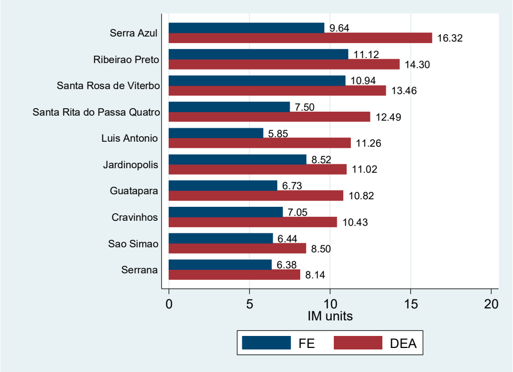

Graph S1.13.4 - Potential years of life gained for Central Araraquara (RRAS13), FE x DEA.

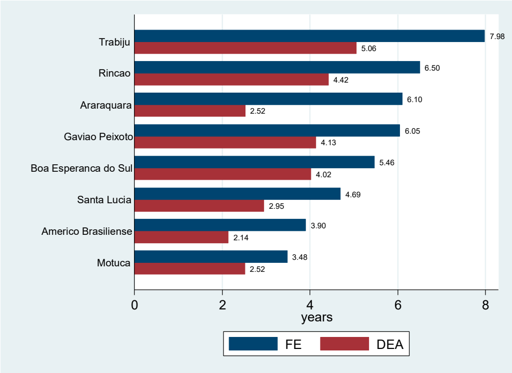

Graph S2.13.4- Potential reduction in IM rates for Central Araraquara (RAS13), FE x DEA

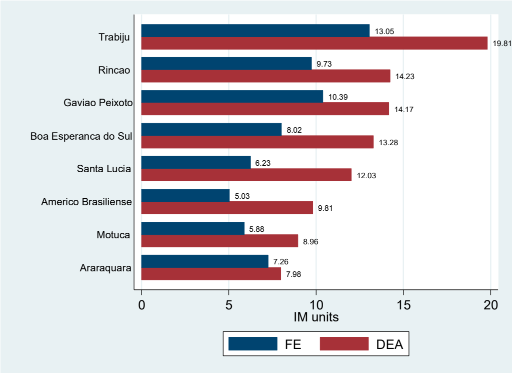

Graph S1.13.5 - Potential years of life gained for Centro Oeste Araraquara (RRAS13), FE x DEA.

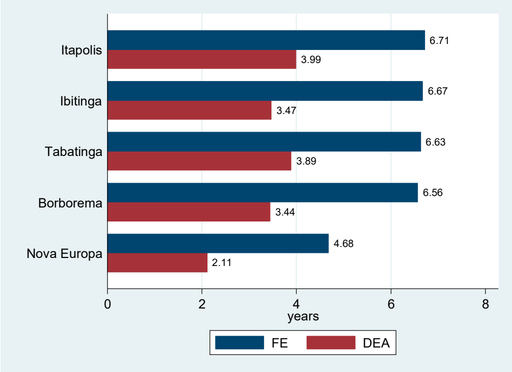

Graph S2.13.5- Potential reduction in IM rates for Centro Oeste Araraquara (RAS13), FE x DEA

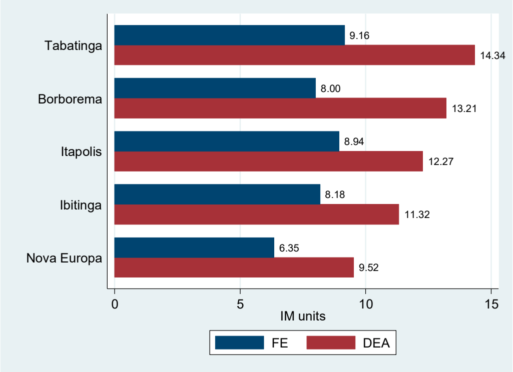

Graph S1.13.6 - Potential years of life gained for Coração Araraquara (RRAS13), FE x DEA.

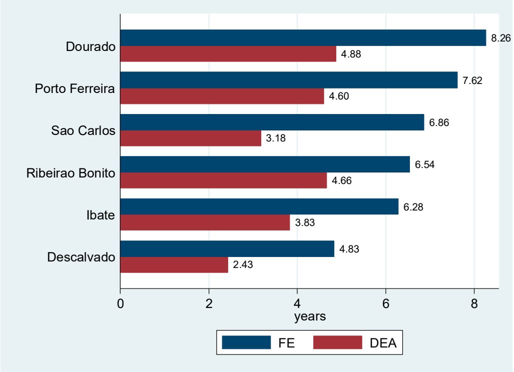

Graph S2.13.6- Potential reduction in IM rates for Coração Araraquara (RAS13), FE x DEA

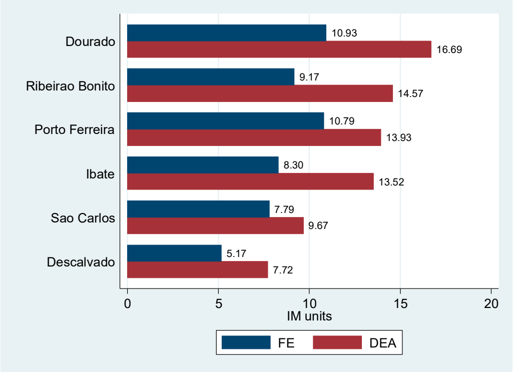

Graph S1.13.7 - Potential years of life gained for Horizonte Verde (RRAS13), FE x DEA.

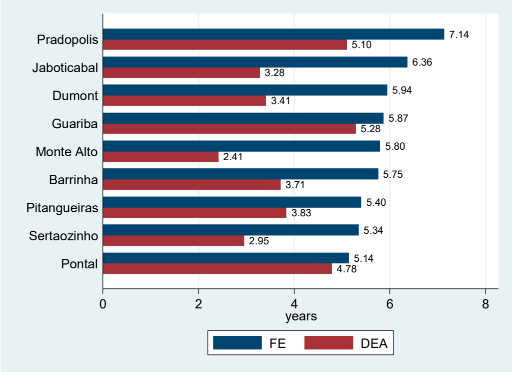

Graph S2.13.7- Potential reduction in IM rates for Horizonte Verde (RAS13), FE x DEA

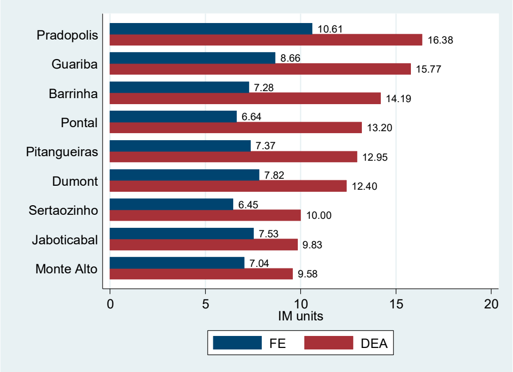

Graph S1.13.8 - Potential years of life gained for Norte Araraquara (RRAS13), FE x DEA.

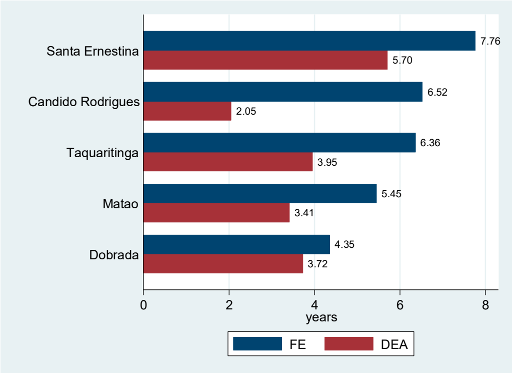

Graph S2.13.8- Potential reduction in IM rates for Norte Araraquara (RAS13), FE x DEA

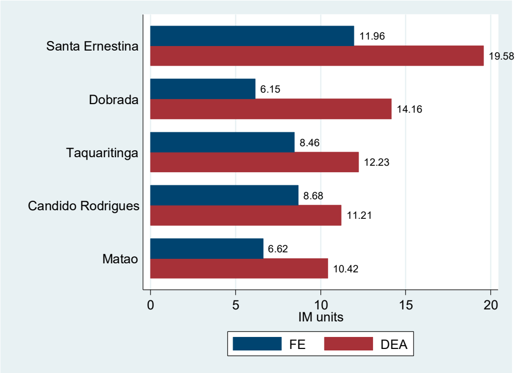

Graph S1.13.9 - Potential years of life gained for Norte Barretos (RRAS13), FE x DEA.

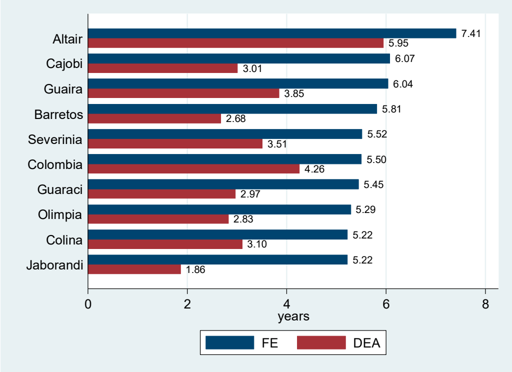

Graph S2.13.9- Potential reduction in IM rates for Norte Barretos (RAS13), FE x DEA

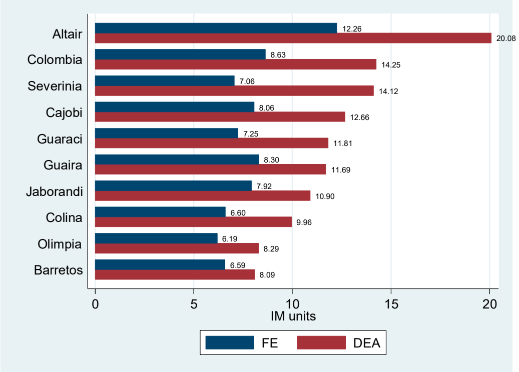

Graph S1.13.10 - Potential years of life gained for Sul Barretos (RRAS13), FE x DEA.

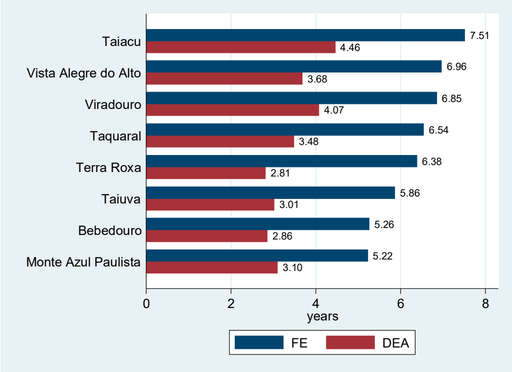

Graph S2.13.10- Potential reduction in IM rates for Sul Barretos (RAS13), FE x DEA

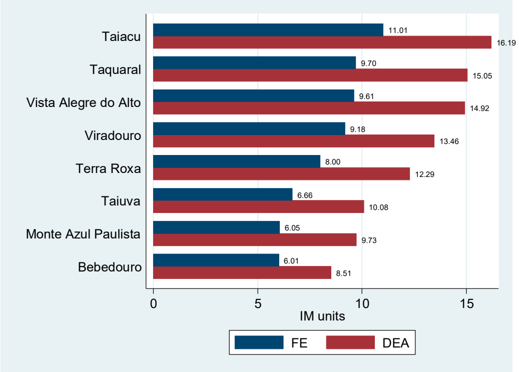

Graph S1.13.11 - Potential years of life gained for Três Colinas (RRAS13), FE x DEA.

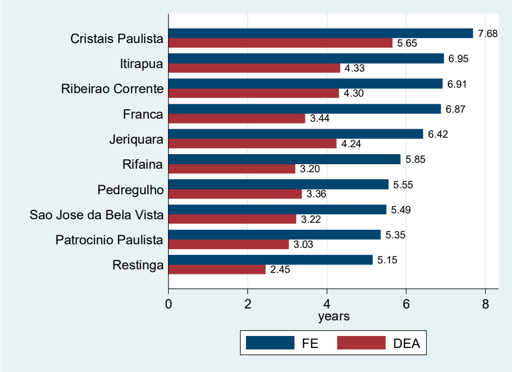

Graph S2.13.11- Potential reduction in IM rates for Três Colinas (RAS13), FE x DEA

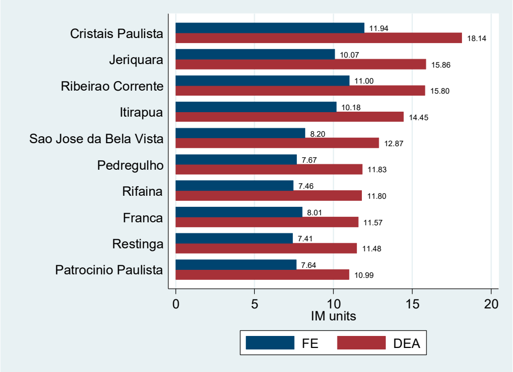

Graph S1.13.12 - Potential years of life gained for Vale das Cachoeiras (RRAS13), FE x DEA.

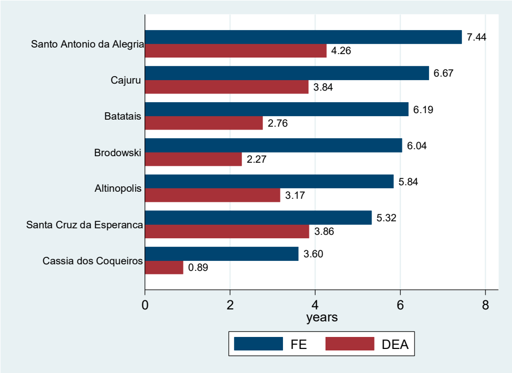

Graph S2.13.12- Potential reduction in IM rates for Vale das Cachoeiras (RAS13), FE x DEA

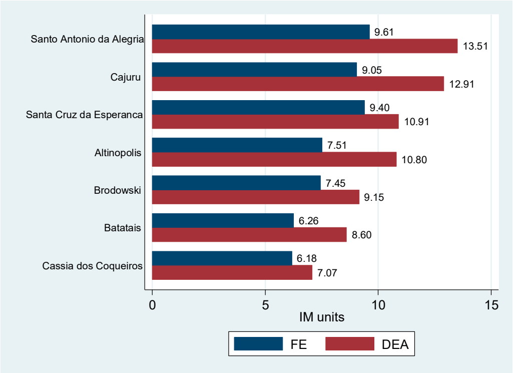

Graph S1.14.1 - Potential years of life gained for Araras (RRAS14), FE x DEA.

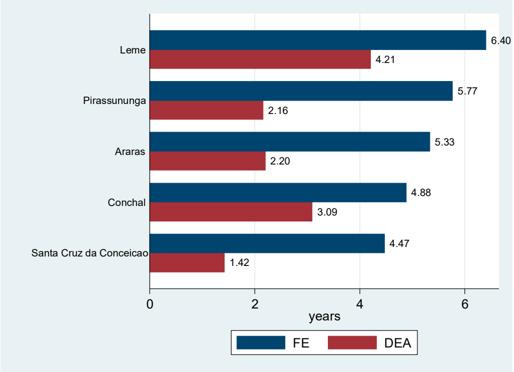

Graph S2.14.1- Potential reduction in IM rates for Araras (RAS14), FE x DEA

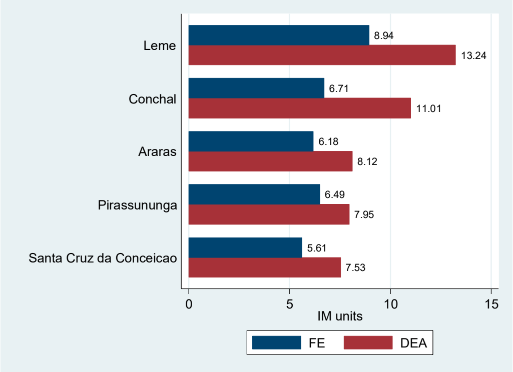

Graph S1.14.2 - Potential years of life gained for Limeira (RRAS14), FE x DEA.

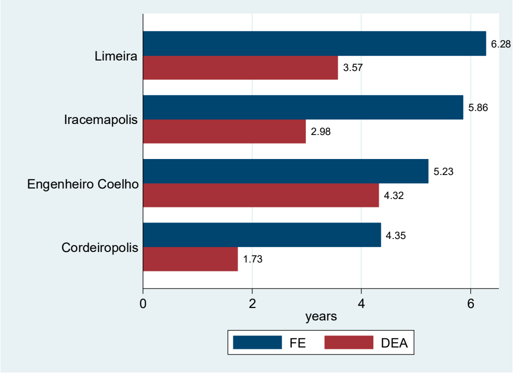

Graph S2.14.2- Potential reduction in IM rates for Limeira (RAS14), FE x DEA

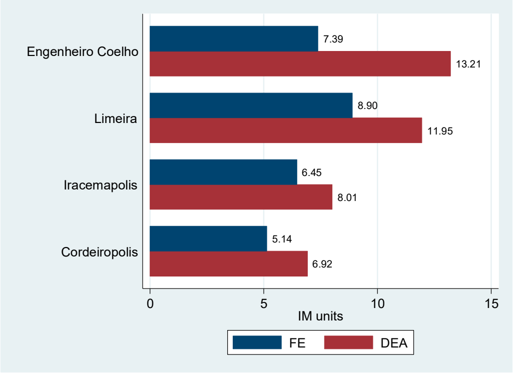

Graph S1.14.3 - Potential years of life gained for Piracicaba (RRAS14), FE x DEA.

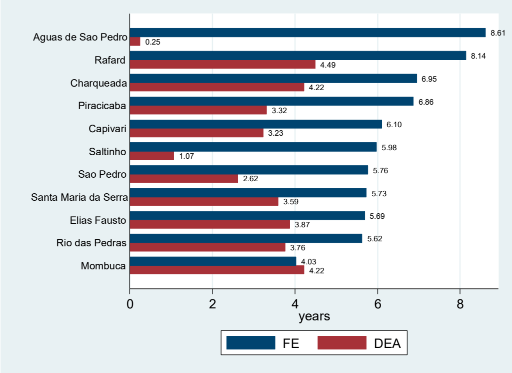

Graph S2.14.3- Potential reduction in IM rates for Piracicaba (RAS14), FE x DEA

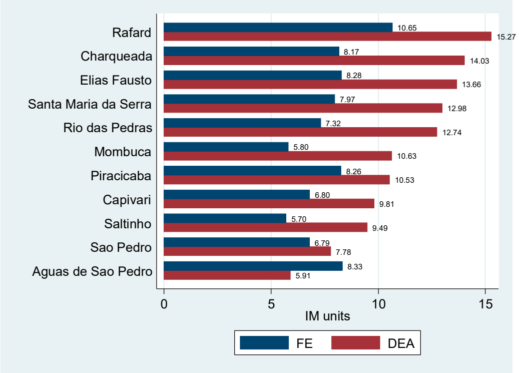

Graph S1.14.4 - Potential years of life gained for Rio Claro (RRAS14), FE x DEA.

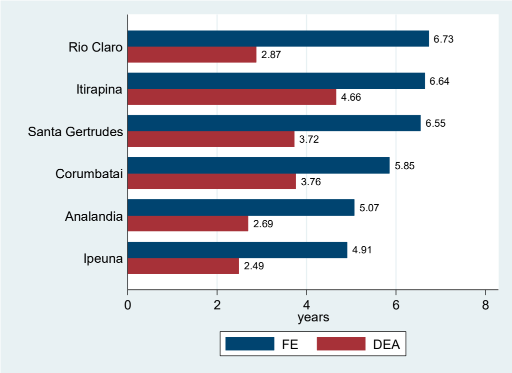

Graph S2.14.4- Potential reduction in IM rates for Rio Claro (RAS14), FE x DEA

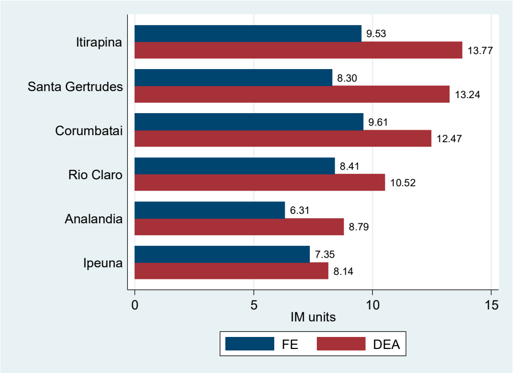

Graph S1.15.1 - Potential years of life gained for Baixa Mogiana (RRAS15), FE x DEA.

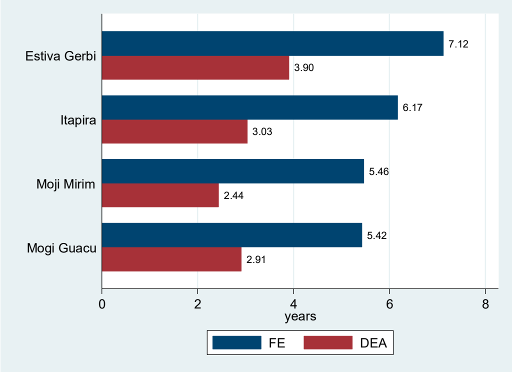

Graph S2.15.1- Potential reduction in IM rates for Baixa Mogiana (RAS15), FE x DEA

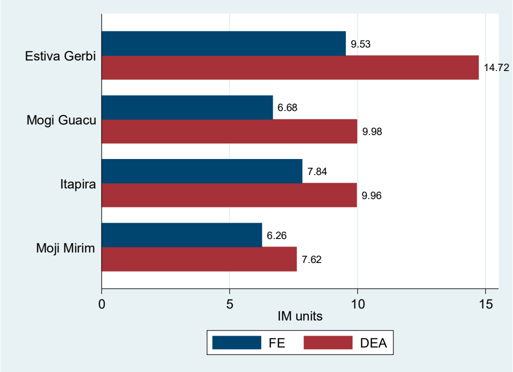

Graph S1.15.2 - Potential years of life gained for Circuito das Águas (RRAS15), FE x DEA.

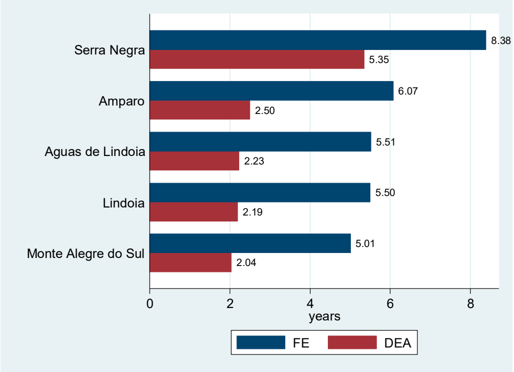

Graph S2.15.2- Potential reduction in IM rates for Circuito das Águas (RAS15), FE x DEA

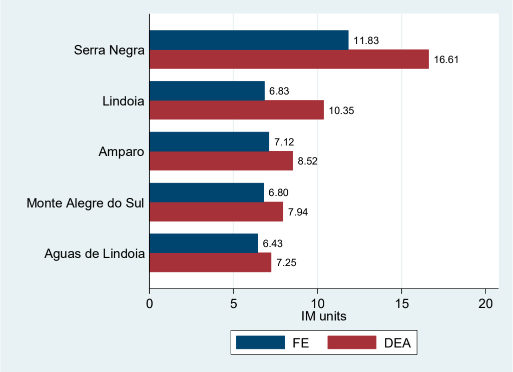

Graph S1.15.3 - Potential years of life gained for Mantiqueira (RRAS15), FE x DEA.

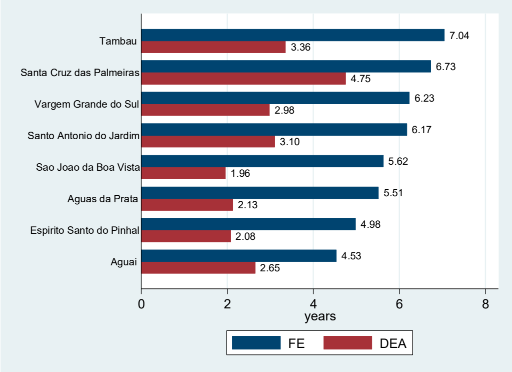

Graph S2.15.3- Potential reduction in IM rates for Mantiqueira (RAS15), FE x DEA

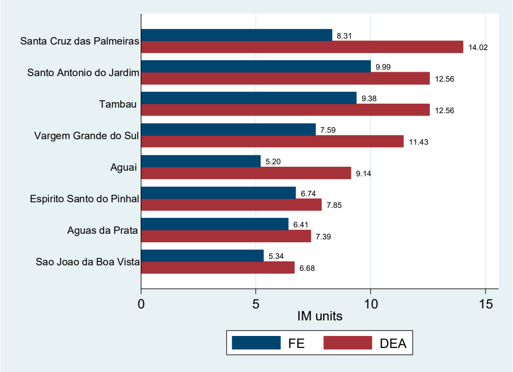

Graph S1.15.4 - Potential years of life gained for Região Metropolitana de Campinas (RRAS15), FE x DEA.

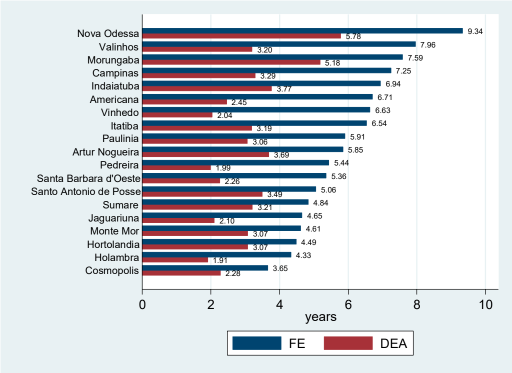

Graph S2.15.4- Potential reduction in IM rates for Região Metropolitana de Campinas (RAS15), FE x DEA

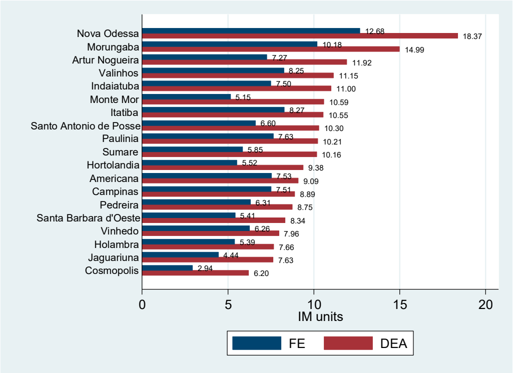

Graph S1.15.5 - Potential years of life gained for Rio Pardo (RRAS15), FE x DEA.

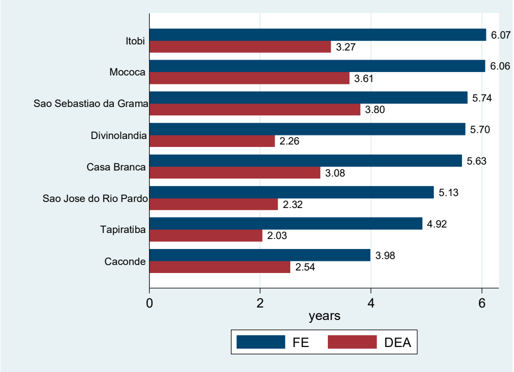

Graph S2.15.5- Potential reduction in IM rates for Rio Pardo (RAS15), FE x DEA

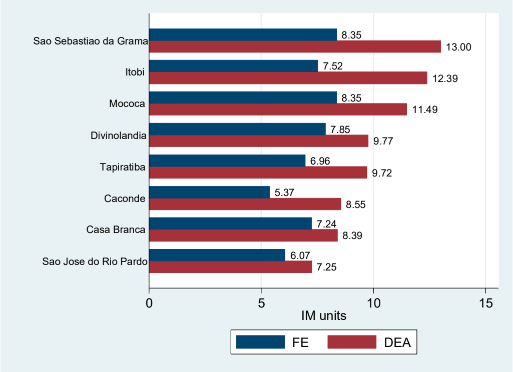

Graph S1.16 - Potential years of life gained for Bragança and Jundiaí (RRAS16), FE x DEA.

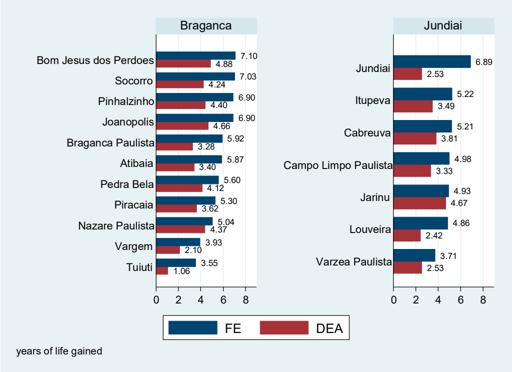

Graph S2.16- Potential reduction in IM rates for Bragança and Jundiaí (RAS16), FE x DEA

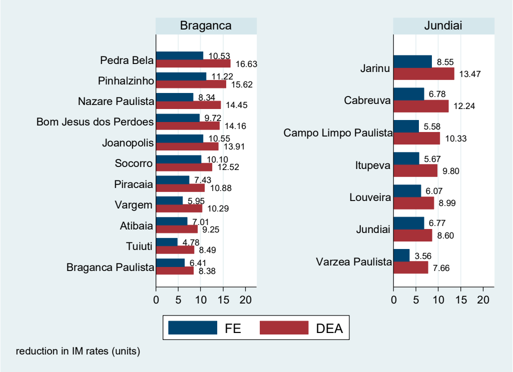

Graph S1.17.1 - Potential years of life gained for Alto Vale do Paraíba (RRAS17, FE x DEA.

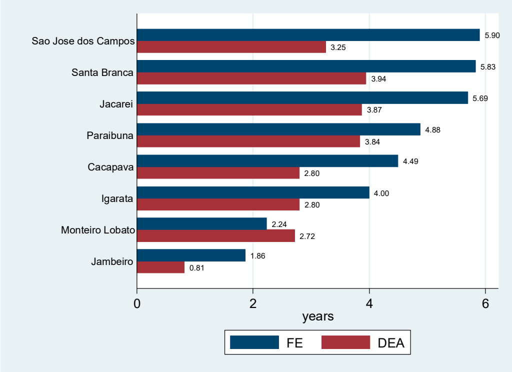

Graph S2.17.1- Potential reduction in IM rates for Alto Vale do Paraíba (RAS17), FE x DEA

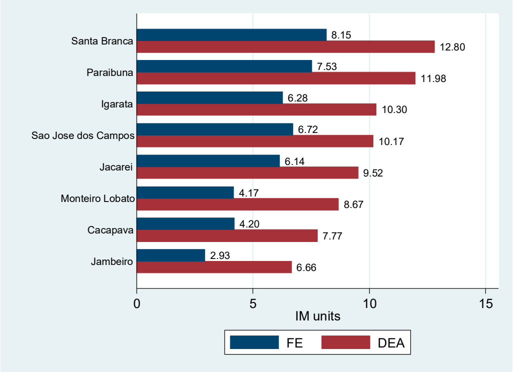

Graph S1.17.2 - Potential years of life gained for Circuito da Fé – Vale Histórico (RRAS17, FE x DEA.

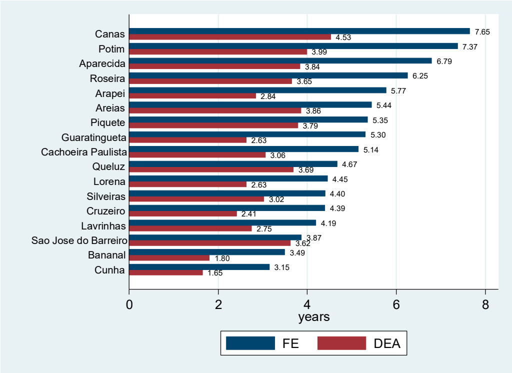

Graph S2.17.2- Potential reduction in IM rates for Circuito da Fé – Vale Histórico (RAS17), FE x DEA

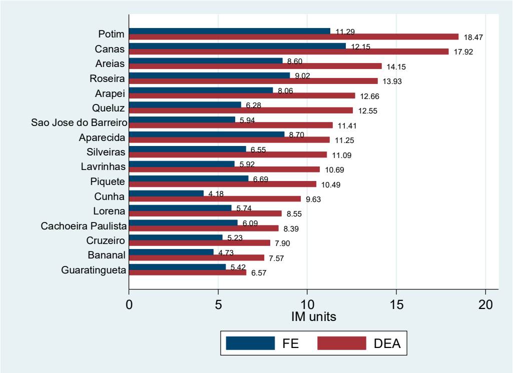

Graph S1.17.3 - Potential years of life gained for Litoral Norte (RRAS17, FE x DEA.

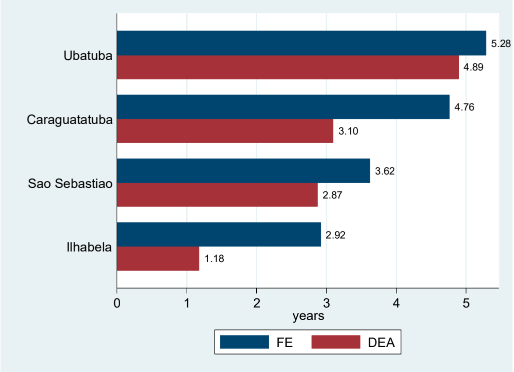

Graph S2.17.3- Potential reduction in IM rates for Litoral Norte (RAS17), FE x DEA

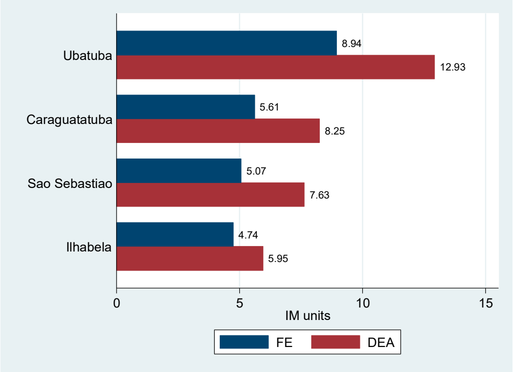

Graph S1.17.4 - Potential years of life gained for Vale do Paraíba-Região Serrana (RRAS17, FE x DEA.

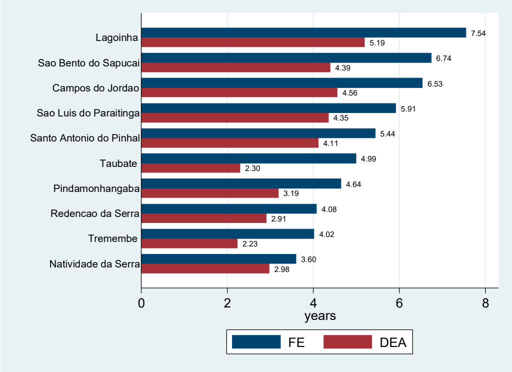

Graph S2.17.4- Potential reduction in IM rates for Vale do Paraíba-Região Serrana (RRAS17), FE x DEA

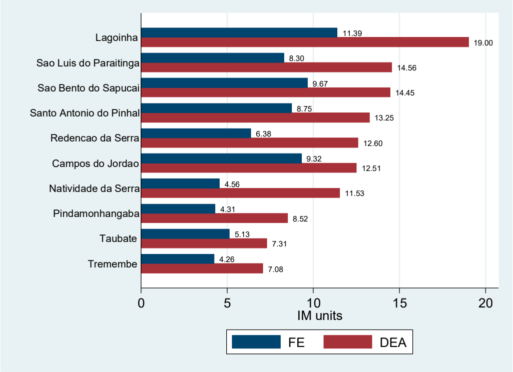

Supplement: Supplementary file 1 [file ijerph-19-02990-s001.zip › ijerph-1546916-supplementary.pdf]
